# Analyzing the role of cancer‐associated fibroblast activation on macrophage polarization

**DOI:** 10.1002/1878-0261.13454

**Published:** 2023-06-07

**Authors:** Marina Bruch‐Oms, Rubén Olivera‐Salguero, Rocco Mazzolini, Beatriz del Valle‐Pérez, Paula Mayo‐González, Ángel Beteta, Raúl Peña, Antonio García de Herreros

**Affiliations:** ^1^ Cancer Research Program, Unidad Asociada al CSIC Institut Hospital del Mar d'Investigacions Mèdiques (IMIM) Barcelona Spain; ^2^ Department of Medicine and Life Sciences Universitat Pompeu Fabra Barcelona Spain; ^3^ Applied Metabolomics Research Laboratory Institut Hospital del Mar d'Investigacions Mèdiques (IMIM) Barcelona Spain

**Keywords:** CAF, macrophage polarization, Prostaglandin E_2_, Snail1, TGFβ

## Abstract

Snail1 is a transcriptional factor required for cancer‐associated fibroblast (CAF) activation, and mainly detected in CAFs in human tumors. In the mouse mammary tumor virus‐polyoma middle tumor‐antigen (MMTV‐PyMT) model of murine mammary gland tumors, *Snai1* gene deletion, besides increasing tumor‐free lifespan, altered macrophage differentiation, with fewer expressing low levels of MHC class II. Snail1 was not expressed in macrophages, and *in vitro* polarization with interleukin‐4 (IL4) or interferon‐γ (IFNγ) was not altered by *Snai1* gene depletion. We verified that CAF activation modified polarization of naïve bone‐marrow‐derived macrophages (BMDMΦs). When BMDMΦs were incubated with Snail1‐expressing (active) CAFs or with conditioned medium derived from these cells, they exhibited a lower cytotoxic capability than when incubated with Snail1‐deleted (inactive) CAFs. Gene expression analysis of BMDMΦs polarized by conditioned medium from wild‐type or Snai1‐deleted CAFs revealed that active CAFs differentially stimulated a complex combination of genes comprising genes that are normally induced by IL4, downregulated by IFNγ, or not altered during the two canonical differentiations. Levels of RNAs relating to this CAF‐induced alternative polarization were sensitive to inhibitors of factors specifically released by active CAFs, such as prostaglandin E_2_ and TGFβ. Finally, CAF‐polarized macrophages promoted the activation of the immunosuppressive regulatory T cells (T‐regs). Our results imply that an active CAF‐rich tumor microenvironment induces the polarization of macrophages to an immunosuppressive phenotype, preventing the macrophage cytotoxic activity on tumor cells and enhancing the activation of T‐reg cells.

## Graphical abstract

When activated by tumor‐derived factors in a Snail1‐dependent fashion, cancer‐activated fibroblasts secrete PGE_2_ and TGFβ and induce the polarization of macrophages to an alternative MHCII‐low state, preventing their cytotoxic activity on tumor cells and promoting immune evasion by the activation of regulatory T immune‐suppressive cells.

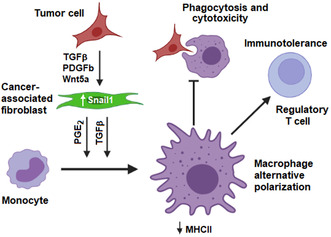

**Abbreviations**
Arg1: arginase 1
Arg2: arginase 2
BMDMΦ: bone‐marrow‐derived macrophage
BSA: bovine seroalbumin
CAF: cancer‐associated fibroblasts
CM: conditioned medium
DFMO: difluoromethylornithine
DMEM: Dulbecco Modified Eagles' Medium
EMT: epithelial‐to‐mesenchymal transition
FBS: fetal bovine serum
GEO: Gene Expression Omnibus
HMEC: human microvascular endothelial cells
IFNγ: interferon‐γ
IL4: interleukin‐4
KO: *Snai1* KO cells
MEF: murine embryo fibroblasts
MMTV: mouse mammary tumor virus
MSC: mesenchymal stem cells
Nos2: inducible nitric oxide synthase
OPN: osteopontin
OVA: ovalbumin
PBS: phosphate‐buffered saline
PDMΦ: peritoneum‐derived macrophage
PGE_2_
: prostaglandin E_2_
PyMT: polyoma middle T antigen
qRT‐PCR: real‐time quantitative PCR coupled to retrotranscription
S3I: S3I‐201 Stat3 inhibitor
SB: SB505125 TGFβ receptor inhibitor
TME: tumor microenvironment
T‐regs: regulatory T cells
WT: wild‐type cells
αSMA: α‐smooth muscle actin

## 1 Introduction

The transcriptional factor Snail1 was initially described as an inducer of the epithelial‐to‐mesenchymal transition (EMT) [1, 2], but it has also been demonstrated that it is essential for the activation of fibroblasts [3, 4]. In EMT and fibroblast activation, Snail1 acts both as a transcriptional repressor and as a transcriptional activator and is required for the expression of mesenchymal genes [5, 6]. Accordingly, Snail1 depletion in cancer‐associated fibroblasts (CAF) or in other mesenchymal cells decreases the expression of vimentin, fibronectin, α‐smooth muscle actin (αSMA) and other CAF markers [3, 4]. Snail1 depletion in these cells also inhibits (a) their migration and invasion in response to several cytokines and growth factors, and (b) the deposition and physical characteristics of the extracellular matrix [3, 7, 8, 9]. Moreover, Snail1‐expressing CAFs secrete factors, such as prostaglandin E_2_ (PGE_2_) and TGFβ, that enhance the migration and invasion of epithelial and mesenchymal cells placed in their vicinity [7, 8].

Expression of Snail1 has been analyzed in multiple tumors and has been associated with a poor prognosis [10]. In most neoplasms, Snail1 is predominantly detected in CAFs although some invasive tumoral epithelial cells also express this factor (for instance, see [11]). CAFs act on tumor development and metastasis affecting many cancer hallmarks [12]. Although several types of CAFs have been reported on the basis of their transcriptional profile [13], *in vitro* Snail1 is required for the expression of the genes used for their characterization [3, 4, 7, 8, 9]. Moreover, Snail1 depletion in tumor CAFs also prevents cellular functions associated with activation, as an increased invasion [3, 4, 7, 8, 9]; thus, Snail1 is considered essential for CAF activation. Accordingly, *Snai1* gene elimination in adult mice retards tumor development and prevents metastasis [8, 14].

Macrophages are the most abundant immune cells in the tumor microenvironment (TME) [15]. Macrophages are not a single population but rather a collection of cell types with different functions. Traditionally, polarized macrophages have been classified as the classically activated M1 macrophages, which express high levels of MHC class II molecules and other markers such as inducible nitric oxide synthase (Nos2) and possess tumoricidal activity; and the alternatively activated M2 macrophages which have low expression of MHCII, high levels of Arginase 1 (Arg1), and favor tumor progression [15]. These two states are obtained *in vitro* upon treatment with interferon γ (IFNγ) and interleukin‐4 (IL4), respectively. However, this M1–M2 dichotomy has been considered an oversimplification, as many other intermediate and alternative phenotypes co‐exist within the TME [15, 16]. For instance, some tumor‐derived macrophages can co‐express both archetypical M1 and M2 markers [17]. In general, cytotoxic M1 macrophages are more abundant in early tumor stages and alternatively activated, in advanced tumors; however, both types co‐exist in most neoplasms [18, 19]. Alternative activation of macrophages in tumors has been mainly considered to be triggered by tumor epithelial cells, although some evidences suggest that CAFs also modulate monocyte recruitment and macrophage polarization [15].

Here, we now show how CAF activation modulates macrophage polarization. When analyzing an *in vivo* murine breast tumor model with a deficient activation of CAFs due to Snail1 depletion, we realized that the total number of macrophages in mammary gland tumors was not different in both conditions but Snail1‐depleted tumors had a misbalanced polarization, with more MHCII‐high, classically activated macrophages. To definitively demonstrate that CAF activation is responsible for this alteration, we polarized naïve bone‐marrow‐derived macrophages (BMDMΦs) using either active CAFs or active CAFs conditioned medium. Compared with CAFs with a *Snai1* knockout (KO), which are therefore deficient in activation, wild‐type (WT) CAFs induced the polarization of macrophages to a state with lower cytotoxic activity. By analyzing the genes specifically expressed by these alternatively polarized macrophages, we have now identified active CAF‐derived factors responsible for this polarization.

## 2 Materials and methods

### 2.1 Antibodies and reagents

The antibodies used in this article are listed in Table S1. The following reagents were also used: prostaglandin E2 (PGE_2_; Cayman Chemicals, Ann Arbor, MI, USA, 14010), TGFβ (Peprotech, Cranbury, NJ, USA, 100‐21), IL4 and IFNγ (both from Immunotools GmbH, Friesoythe, Germany, 11340045 and 12343534), IL6 (Prospec, Rehovot, Israel, HZ‐1019), osteopontin (OPN, BioLegend, San Diego, CA, USA, 763602), TGFβ Receptor inhibitor SB505125 (S4696), prostaglandin EP2 Receptor inhibitor L‐161982 (SML‐0690), prostaglandin EP4 Receptor inhibitor PF‐04418948 (PZ0213), difluoromethylornithine (DFMO, D193‐25MG), celecoxib (70008, all five from Sigma‐Aldrich, Saint Louis, MO, USA), and the Stat3 inhibitor S3i‐201 (Selleckchem, Houston, TX, USA, S1155).

### 2.2 Cell culture

Wild‐type (WT) CAF from PyMT tumors (see Section 2.12), and murine embryo fibroblasts (MEF), either WT or knockout (KO) for *SNAI1* gene, were previously established in our laboratory [4, 8]. Authenticated NMuMG (Research Resource Identifier CVCL_0075) and MCF7 (CVCL_0031) cells were obtained from IMIM Cell Bank; authenticated human microvascular endothelial cells (HMEC1, CVCL_0307) were a kind gift of Dr MI Díaz‐Ricart (Hospital Clínic, Barcelona, Spain). Cell line authentication was performed by American Type Culture Collection, Manassas, VI, USA. The generation of mesenchymal stem cells (MSCs), epithelial PyMT tumor AT3 and BTE136 cells, and HT‐29M6 (CVCL_G077) transfected with Snail1 has also been described [1, 8, 9]. All cell lines were cultured in Dulbecco's Modified Eagle's Medium (DMEM, Gibco, Grand Island, NY, USA) supplemented with 10% fetal bovine serum (FBS, Gibco) at 37 °C in a humidified incubator (Heracell™ 150, Heracell, Thermo Scientific, Waltham, MA, USA) with 5% CO_2_ and were periodically tested to verify that they remained mycoplasma‐free.

### 2.3 Macrophage isolation and culture

To obtain bone‐marrow‐derived macrophages (BMDMΦs), male C57Bl/6J 8–12‐week‐old mice (Jackson Laboratories, Bar Harbor, ME, USA) were sacrificed and the femoral and tibial marrow was flushed with a 25G syringe with complete medium (DMEM 10% FBS, 100 U·mL^−1^ penicillin, 100 μg·mL^−1^ streptomycin, and 2 mm glutamine). Cells were then filtered through a 100 μm mesh and seeded in polystyrene dishes with complete medium supplemented with 25% (vol/vol) L929‐conditioned medium (as a supply of M‐CSF) and incubated for 7 days at 37 °C in 5% CO_2_ atmosphere [20]. At this time, more than 94% of cells were macrophages as assessed by F4/80 staining. Macrophages were then harvested with phosphate‐buffered saline (PBS) plus 5 mm EDTA and plated in cell culture plates for further studies.

Peritoneal macrophages (PDMΦ) were isolated from C57Bl/6J 8–12‐week‐old mice as described in standard protocols [21]. Briefly, mice were injected intraperitoneally with 1 mL of 3% Brewer thioglycolate medium; after 7 days, mice were euthanized and the peritoneal cavity was washed with 10 mL ice‐cold PBS. Peritoneal exudate cells were centrifuged at 400 × ***g*** for 10 min at 4 °C and then cultured in polystyrene dishes with complete medium at 37 °C in 5% CO_2_ atmosphere. Peritoneal cells were sorted by flow cytometry for CD45^+^, CD11b^+^, Ly6G^−^, and F4/80 (high or low) cell surface expression and isolated cells were then processed for analysis of mRNA.

### 2.4 Analysis of macrophages by flow cytometry

Macrophages in suspension were blocked for 5 min in PBS containing an antibody to Fcγ receptors CD16/CD32 (1 μg antibody per 10^6^ cells, BD Pharmingen, San Diego, CA, USA). Cells were then incubated for 20 min in PBS with fluorochrome‐labelled isotype control antibodies or surface marker–specific antibodies (1 μg antibody per 10^6^ cells) and analyzed with a LSRII flow cytometer, facsdiva software (BD Biosciences, Franklin Lakes, NJ, USA) and flowjo V10 software (BD Biosciences). The antibodies used are indicated in Table S1.

### 2.5 Macrophage cytotoxic activity

This activity was determined by three similar assays. First, Tomato‐labelled AT3 or MCF7 cells (1× or 2 × 10^4^ cells, respectively) were seeded with 2 × 10^4^ CAF or MEF (Snai1 WT or KO). After 24 h, 2.25 × 10^4^ macrophages were added to the co‐culture; after 48 h, wells were washed with PBS, and 10 random pictures (10×) were taken to quantify the amount of MCF7 or AT3 Tomato‐labelled cells remaining on the plate. Alternatively, macrophages (2.25 × 10^4^) were incubated with CAF (2 × 10^4^) for 24 h; labelled‐tumor cells (2 × 10^4^) were added; and the number of remaining tumor cells was determined after 48 h as above. Finally, conditioned media (CM) from 2 × 10^4^ CAF cultured in a 6‐well plate with 0.1% FBS for 24 h was diluted with fresh medium (1 : 1) and added to 2 × 10^4^ Tomato‐labelled MCF7 cells with 2.25 × 10^4^ macrophages. Cytotoxicity was calculated as the decrease in the number of labelled cells due to the presence of macrophages; the number of cells without macrophages was used as control.

### 2.6 Macrophage phagocytic activity

BMDMΦs were seeded on 60 mm non‐adherent dishes and treated with DMEM or CAFs CM diluted in DMEM (1 : 1). After 24 h, Tomato‐labelled MCF7 or GFP‐labelled AT3 cells (2.4 × 10^4^) were added for 24 h. Alternatively, GFP‐labelled beads (Fluoresbrite^®^ YG Microspheres, Calibration Grade 1.00 μm; Polysciences, Warrington, PA, USA; 3.8 × 10^6^ particles) were added to the polarized cells for 30 min. Cells were harvested with PBS plus 5 mm EDTA and stained as described in flow cytometry with PE anti‐mouse CD45. Tomato and GFP fluorescence were analyzed with a Fortessa and LSRII flow cytometer, facsdiva software (BD Biosciences), and flowjo V10 software (BD Biosciences).

### 2.7 Analysis of lymphocyte activation by flow cytometry

Mice splenocytes were isolated from C57Bl/6J 8–12‐week‐old mice spleen. Briefly, mice were sacrificed, and spleen was resected and put on a 100 μm cell strainer. Spleen was disaggregated on the cell strainer with a syringe plunger and 30 mL of PBS plus 0.1% BSA. Cells were centrifuged at 500 ***g*** for 5 min. Next, the pellet was resuspended with 8 mL of ACK buffer (Gibco) to disrupt all the erythrocytes and left at 37 °C for 5 min. To inactivate ACK buffer, 1 : 1 RPMI 1640 media was added and the mix was centrifuged. The pellet was resuspended with 10 mL of PBS (0.1% BSA) and transferred to a new Falcon tube with a 70 μm cell strainer. Cells were centrifuged at 500 ***g*** for 5 min, and the pellet was resuspended with 10 mL of the activation medium, CM from polarized macrophages, DMEM (5% FBS), or CAFs CM and left for 24 h. Lymphocytes were pelleted down, and 1 × 10^6^ cells were resuspended in 100 μL of ice‐cold PBS. Cells were incubated in the dark for 20 min in PBS with surface marker‐specific antibodies CD3‐BV510, CD45‐FITC, CD8‐PE‐Cy7, and CD69‐PE (1 μg antibody per 10^6^ cells) and washed with 1 mL of ice‐cold PBS followed by a centrifugation at 500 ***g*** for 5 min. Supernatants were discarded, and pellets were resuspended with 100 μL of ice‐cold PBS with DAPI (1 : 10 000), if needed. After incubation for 5 min in the darkness, a final wash was performed.

For regulatory T cells (T‐reg) staining, a variation of this protocol was used. Live/dead marker was added for 5 min in the dark (1 : 1000). Next, cells were washed with PBS and pelleted down at 500 ***g*** for 5 min. For the intracellular staining, Fix & Perm Cell Permeabilization Kit (GAS004, Life Technologies, Carlsbad, CA, USA) was used. Thus, the cell pellet was resuspended in 100 μL of reagent A together with CD3‐APC‐Cy7 (0.5 μg antibody per 10^6^ cells), CD25‐APC, and CD4‐PerCP (1 μg antibody per 10^6^ cells) and cells were incubated in the dark for 20 min. Then, cells were washed with PBS, centrifuged, and pellet was resuspended in 100 μL of reagent B with FoxP3‐PE antibody (0.5 μg antibody per 10^6^ cells) and incubated for 20 min in the dark before a final wash.

Samples were resuspended with at least 100 μL of PBS and analyzed with a LSRII flow cytometer, facsdiva software (BD Biosciences), and flowjo V10 software (BD Biosciences).

### 2.8 Lymphocyte cytotoxic activity

For lymphocyte cytotoxicity assay, CD8^+^ T cells from OT‐I mouse model were obtained as described in Section 2.7. OT‐I CD8 T cells specifically recognize Ovalbumin (OVA) peptide 257–264 (SIINFEKL). Around 1 × 10^6^ cells were treated with fresh RPMI medium supplemented 10% FBS and with conditioned medium from polarized macrophages, DMEM plus 5% FBS, or CAF CM (in all cases 1 : 2) for 24 h. Then, 1 × 10^4^ cells were cocultured (1 : 1) with AT3‐OVA tumor cells (a kind gift of T. Celià‐Terrassa and I. Pérez, IMIM, Barcelona) for 72 h in RPMI plus 10% FBS. Cell viability was determined after 72 h staining with Crystal Violet.

### 2.9 Analysis of cytokine secretion by CAFs

CAF (Snail1 WT or KO) were maintained in regular culture medium without FBS overnight. The analysis of the conditioned media was performed using Mouse L308 Array (RayBiotech L‐Series, Peachtyree Corners, GA, USA, AAM‐BLM‐1B‐2) according to the manufacturer's instructions. After developing the membranes, the signal was quantified by Protein Array Analyzer for imagej (National Institute of Health, Bethesda, MY, USA). Prostaglandin E_2_ (PGE_2_) and TGFβ levels were determined by ELISA (Direct Biotrack Assay, RPN222, GE Healthcare Life Sciences, Chicago, IL, USA, for PGE_2_, and DB‐100B, R&D Systems, Minneapolis, MN, USA, for TGFβ) in 2‐day‐conditioned medium from WT or Snail1 KO CAFs.

### 2.10 RNA microarray analysis

BMDMΦs were treated with either complete medium, conditioned medium of 24 h serum‐starved CAFs (*Snai1* WT or *Snai1* KO), IL4, or IFNγ for 48 h. Gene‐expression profiling of treated BMDMΦs was assessed using Clariom‐S, Mouse microarray (Affymetrix, Santa Clara, CA, USA). Duplicates of each condition were processed according to the following Affymetrix protocols: GeneChip WT PLUS Reagent kit (P/N 703174 2017) and Expression Wash, Stain and Scan User Manual (P/N 702731 2017; Affymetrix Inc.). After data normalization and standard QC, differential gene expression between sample signals was analyzed with transcriptome analysis console (TAC) Software (Thermo). A complete list of the genes induced in the different conditions has been deposited in NCBI's Gene Expression Omnibus (GEO) (GSE206404, token sbsxciochrmpnon). Gene set enrichment analysis was performed with gsea (v4.1.0) software against April 2021 datasets. Datasets were considered statistically positive when FDR > 25% and *P* value < 0.05.

### 2.11 Human tumor bioinformatic analysis

The expression data of mRNA were obtained from TCGA Breast Invasive Carcinoma (PanCancer Atlas) data in the cBioPortal public database (http://www.cbioportal.org/) in May 2021. In this study, we assessed the mRNA expression of macrophages signature (*PLXDC2*, *TIMP1*, *CCR5*, *ARG2*, *CD33*, *CXCR4*, *MRC1*, *CCR1* and *ARG1*) and *SNAI1*, *FN1*, *ACTA2*, *SPP1*, *COL1A1*, *VIM*, *PECAM1*, *VEGFA*, *FGF2*, *FOXP3*, and *IKZF2*.

The analysis of single‐cell gene expression in breast tumors was performed from data published in [22]. Genes corresponding to secreted proteins showing more than a threefold increase in CAF WT versus CAF Snail1 KO (see below) were considered for the analysis of CAF populations. For myeloid populations, the 25 genes presenting a higher stimulation in macrophages polarized with CAF WT conditioned medium versus CAF Snail1 KO polarized cells were used. The association between the percentage of different categories of CAF (with respect to total CAFs) and myeloid cells (with respect to total myeloid cells) was also determined. Samples were analyzed online at https://singlecell.broadinstitute.org/single_cell/portal (Broad Institute, Harvard University, USA).

### 2.12 Animals

Animals were housed in a specific pathogen‐free area and fed *ad libitum* at the Parc de Recerca Biomèdica de Barcelona (PRBB) Animal Facility. All the animal procedures were previously approved by the Animal Research Ethical Committee from the PRBB and by the Generalitat de Catalunya (CEEA AGH‐19‐0028) following the EU Directive 2010/63/EU. We have previously described [4] the generation of a murine line with *Snai1* floxed (Snail1^Flox^), and *Snai1* wild‐type (Snail1^+^) or *Snai1* deleted (Snail1^−^) alleles and a Cre recombinase‐estrogen receptor fusion gene under the control of the *β‐Actin* promoter (β‐Actin Cre‐ER). These animals were mated with MMTV‐PyMT mice which develop spontaneously Luminal B breast cancer tumors [23]. This murine line expresses the polyoma Virus middle T antigen (PyMT) under the control of the mouse mammary tumor virus promoter (MMTV); female mice develop mammary tumors with lung metastases. Depletion of Snail1 in MMTV‐PyMT, β‐Actin Cre‐ER, *Snai1* ^Flox/−^ (or *Snai1* ^Flox/+^ as control) was performed by five intraperitoneal daily doses of tamoxifen injection (0.2 mg·g^−1^) as described [8] in 8‐week‐old mice; an additional dose was administrated every 3 weeks until animals were euthanized. Mice were palpated twice per week to determine the tumor onset and tumor size was determined using a caliper. When tumors reached 4 cm^3^, animals were sacrificed.

### 2.13 Subcutaneous injection of tumor cells

For tumorigenesis assays, 8‐ to 12‐week‐old NSG mice (Jackson Laboratories) were subcutaneously inoculated on their hind leg flanks with 1 × 10^5^ cells per cell type of AT3 or AT3 plus polarized macrophages on each flank, following the approved protocol. After 3 weeks, animals were euthanized, and tumors were collected in ice‐cold PBS. Then, tissues were measured and weighed. During tumorigenesis assays, mice were monitored twice a week for tumor incidence and animal well‐being.

### 2.14 Determination of spermidine in macrophages

Spermidine was determined in macrophages by liquid chromatography coupled to tandem mass spectrometry (LC–MS/MS) using a calibration external curve (0.5–100 ng·mL^−1^). Macrophages were lysed, homogenized, and stored at −20 °C to their analysis. Twenty‐five microliter of the lysed cell homogenate and 25 μL of an internal standard solution containing spermidine‐d8 were transferred into a glass tube and evaporated to dryness under nitrogen stream. Then, 100 μL of 2 m Na_2_CO_3_ buffer (pH 11) and 100 μL of dansyl chloride (10 mg·mL^−1^ in acetone) were transferred into the glass tube for derivatization. The resulting mixture was vortexed (60 s), centrifugated (5 min, 3000 ***g***, room temperature), and incubated (60 °C, 2 h). After a new centrifugation at the same conditions, the mixture was evaporated to dryness and reconstituted with 100 μL of a solution methanol: water (1 : 1). Finally, 8 μL of the extract was injected into the LC–MS/MS system. Spermidine was quantified by the ion transition 845 > 170. masslynx software V4.1 (Waters Associates, Milford, MA, USA) was used for peak integration and data management.

### 2.15 Reverse transcription and real‐time quantitative PCR

Total RNA was extracted using GenElute TM Mammalian Total RNA Miniprep Kit (Sigma). Expression levels of transcripts were calculated by real‐time quantitative PCR coupled to retrotranscription (RT‐PCR), using the Transcriptor First Strand cDNA Synthesis kit (Roche, Basel, Switzerland) and the LightCycler 480 Real‐Time PCR System (Roche). RNA levels were determined in triplicate. Reactions were performed using the primers listed in Table S2.

### 2.16 Cell lysis and protein analysis by western blot

Cell extracts were obtained in SDS lysis buffer (Tris‐HCl pH 7.4, 50 mm; SDS 2% and glycerol 10%) and analyzed by western blot using the indicated primary antibodies (Table S1) and HRP‐conjugated secondary antibodies.

### 2.17 Immunohistochemistry

Samples were fixed with p‐formaldehyde (4%) at room temperature. When indicated, consecutive sections of tumors were used. Fixed samples were dehydrated and paraffin‐embedded according to standard procedures. Sections (2.5 μm) were prepared for immunohistochemical analysis and stained with hematoxylin and eosin for histological evaluation. After standard deparaffination and rehydration of the samples, antigen unmasking was carried immersing the sections in Tris EDTA buffer pH 9 or Citrate buffer pH 6 and boiling for 15 min. Samples were blocked for 2 h in Tris‐buffered saline (TBS) plus FBS (1%) and BSA (1%), and later incubated with Snail1, FoxP3, CD206, or Vimentin antibodies (Table S1). Signal was amplified with EnVision^+^ System HRP Labelled Polymer (anti‐mouse, DAKO, Glostrup, Denmark) and visualized with the DAB kit (DAKO). FoxP3 and Vimentin or CD206 and Vimentin were detected by IHC in consecutive slides. For the analysis of FoxP3‐positive cells, two regions of vimentin high‐ and vimentin low‐staining were manually delimitated on each tumor slide (four animals per group). FoxP3‐positive cells were quantified using imagej software and represented as the percentage of positive cells in the vimentin area.

### 2.18 Cell proliferation

Cell proliferation was measured by adding 0.5 mg·mL^−1^ of 3‐(4,5‐dimethylthiazol‐2‐yl)‐2,5‐diphenyltetrazolium bromide (MTT; Sigma‐Aldrich) in DMEM for 3–4 h at 37 °C. After incubation, cells were solubilized in a solution of DMSO and isopropanol (1 : 4). Absorbance was quantified in an Infinite M200 Microplate Reader (Tecan, Mannedorf, Switzerland) at 590 nm.

### 2.19 Statistical analysis

Data were analyzed by graphpad prism (v6) software (GraphPad Software, Dotmatics, Boston, MA, USA). Tumor‐free graphs were represented by a Kaplan–Meier curve, and *P*‐value was obtained using log‐rank test. In all other experiments, statistical significance was obtained using Student's *t*‐test. For *P*‐values in all the figures: *, *P* < 0.05; **, *P* < 0.01; and ***, *P* < 0.001. In the analysis of expression data in human tumors, the Spearman's rank correlation coefficient or the goodness‐of‐fit test was determined to assess statistical significance.

## 3 Results

### 3.1 *Snai1* genetic depletion decreases the number of MHCII‐low macrophages in the MMTV‐PyMT model of mammary gland tumors

**Fig. 1:**
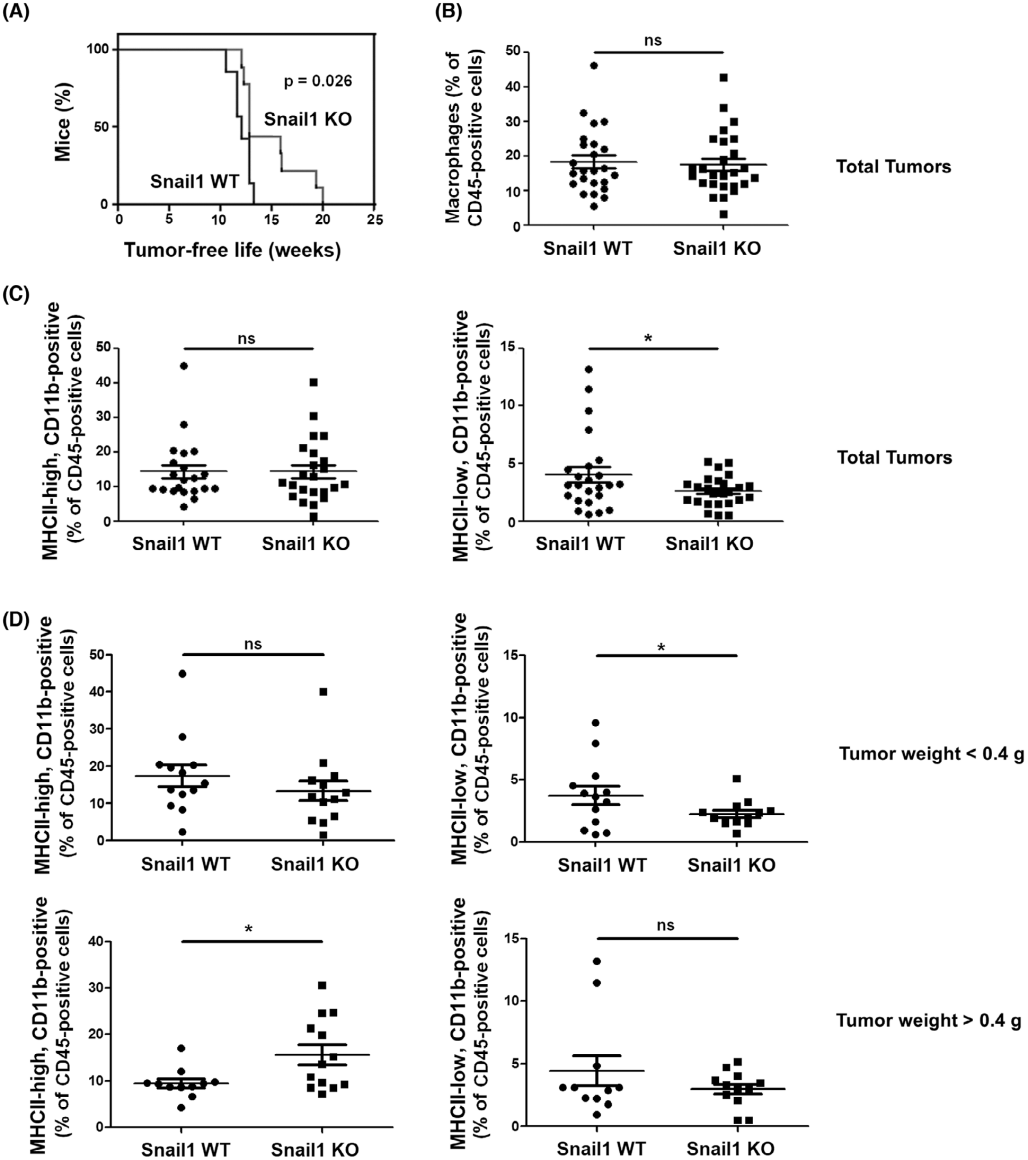
Snail1 deletion alters the ratios of MHCII‐low and ‐high macrophages in MMTV‐PyMT tumors. (A) Tumor‐free life of MMTV‐PyMT mice either Snail1 WT (+/Flox) or Snail KO (−/Flox). Floxed‐Snail1 depletion was performed as indicated in Section 2 when animals were 8 weeks old. Snail1 WT, *n* = 7; Snail KO, *n* = 9. (B, C) Macrophages were purified from tumors using the FACS strategy shown in Fig. S2; macrophages were discriminated by their MHCII expression. (D) Macrophages were obtained from small (less than 0.4 g in weight) or large (more than 0.4 g) tumors. Statistical significance was obtained using Student's *t*‐test. The figures show the average ± SD; ns, not significant; *, *P* < 0.01.

We have generated a murine model with a ubiquitous and inducible depletion of Snail1 gene [4]. These mice were crossed with the MMTV‐PyMT murine line that spontaneously generates mammary gland tumors with a luminal B phenotype [23]. As we and others have previously reported regarding tumor burden [8, 14], depletion of *Snai1* in 6‐week‐old MMTV‐PyMT mice increased their tumor‐free life with respect to mice with WT *Snai1* (Fig. 1A). Snail1 protein expression was mainly observed in stromal cells with a fibroblastic morphology (Fig. S1A), as previously reported [8], although some tumor cells were also positive. Tumors of different sizes were obtained from both types of mice; of note, tumors from WT mice presented a less compact phenotype with a higher stromal infiltration as compared to those from *Snai1* KO mice (Fig. S1B). The macrophage content was determined by cell sorting using the gating strategy shown in Fig. S2. Macrophage number was not significantly different in both genetic backgrounds (Fig. 1B). We also classified macrophages on the basis of their MHCII expression. PyMT‐*Snai1* KO tumors showed fewer MHCII‐low (presumably alternatively activated) macrophages than controls (Fig. 1C). A similar analysis was repeated but classifying the tumors as small (less than 0.4 g of weight) or large (more than 0.4 g). In this case, small tumors from *Snai1* KO mice displayed fewer MHCII‐low macrophages than controls; in contrast, large tumors from these mice contained more MHCII‐high M1 macrophages (Fig. 1D).

**Fig. 2:**
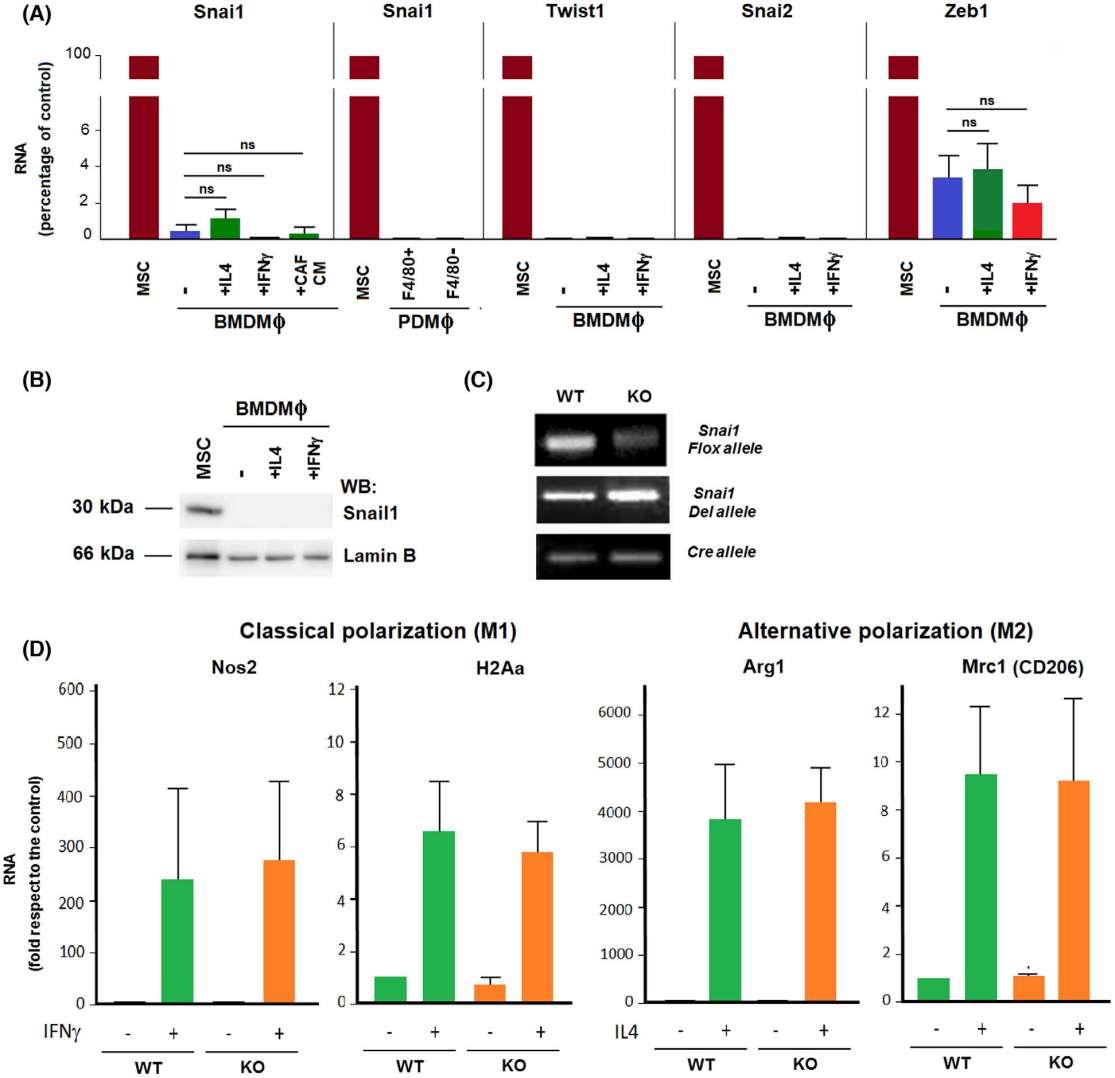
Snail1 is not expressed in macrophages. (A, B) Macrophages were obtained from bone marrow (BMDMΦ) or peritoneum (PDMΦ), and classified as F4/80 high or low. They were activated with IFNγ (100 units·mL^−1^), IL4 (10 ng·mL^−1^) or the conditioned medium from CAF for 24 h. *Snai1*, *Twist1*, *Snai2*, and *Zeb1* RNAs (A) were determined as indicated in Section 2; not‐stimulated mesenchymal stem cells (MSC) were used as reference. The average ± SD of three experiments is shown. Statistical significance was assessed using Student's *t*‐test. (B) Western blot analysis of the samples obtained in (A). (C) DNA from macrophages obtained from Snail1(+/Flox) (WT) or (−/Flox) (KO) mice was analyzed by PCR. Snail1 del or Flox alleles were determined. The results of a representative experiment of three (B) or two (C) performed are shown. (D) RNA was obtained from WT or KO Snail1 BMDMΦ either not stimulated or treated with IFNγ or IL4 for 15 h and expression of M1‐ and M2‐specific genes was assessed by quantitative RT‐PCR (qRT‐PCR). The figures show the average ± SD of four experiments (M1 markers) or ± range of two experiments (M2 markers). Not‐stimulated WT BMDMΦ were used as reference.

We considered the possibility that this altered macrophage differentiation was the consequence of the downregulated Snail1 expression in these cells. However, the levels of *Snai1* RNA detected in bone‐marrow‐derived macrophages (BMDMΦs) were extremely low, less than 1% of the value observed in non‐stimulated mesenchymal stem cells (MSCs; Fig. 2A). Macrophages obtained from the peritoneum presented even lower levels. *Snai1* RNA levels were not substantially increased by *in vitro* polarization of BMDMΦs to classical or alternative phenotypes with IFNγ or IL4, respectively, or by the addition of CAF‐conditioned medium (Fig. 2A; see also below). Western blot analysis also corroborated this lack of Snail1 expression (Fig. 2B). BMDMΦs did not express other transcriptional factors associated with EMT, such as Twist1 or Snail2, and only very low levels of Zeb1 that were much lower than those found in MSCs (around 3%). Finally, *Snai1* genetic depletion in BMDMΦs (Fig. 2C) did not alter their capability to *in vitro* polarize to classical or alternative phenotypes with IFNγ or IL4, as assessed by the expression of *Nos2* and *H2Aa* RNAs (for classical), or *Arg1* and *Mrc1* RNAs (for alternative polarization; Fig. 2D).

These results suggest that the altered polarization of macrophages in PyMT tumors observed in *Snai1* KO mice is the consequence of Snail1 depletion not in these immune cells but in CAFs, which are the cells in which this factor is mainly expressed in tumors. As Snail1 is required for CAF activation, our results also suggest that active and inactive CAFs differ in their capability to polarize macrophages.

### 3.2 Elimination of Snail1 in CAFs impairs their capability to attenuate BMDM cytotoxicity and phagocytosis of tumor cells

**Fig. 3:**
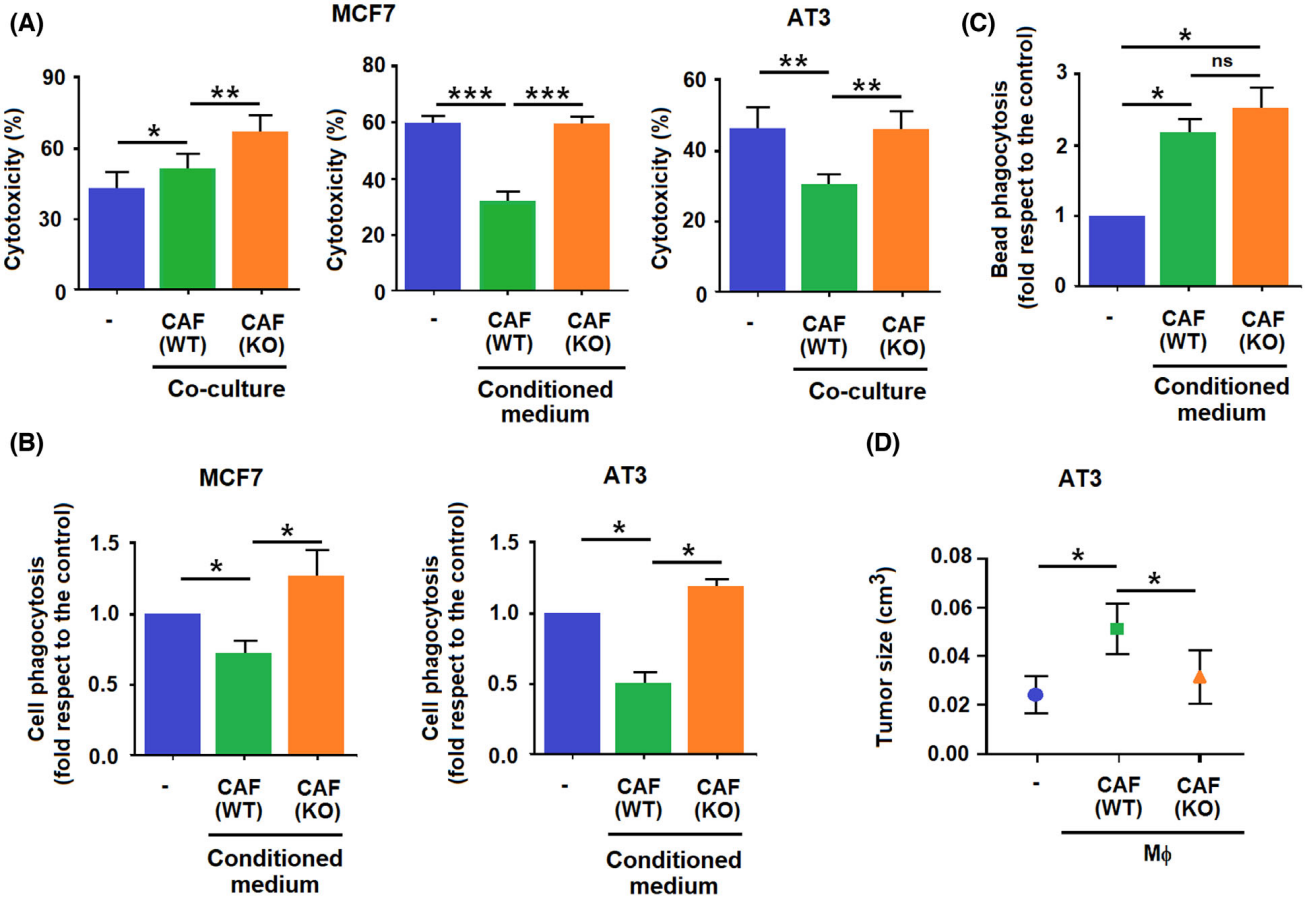
Snail1 depletion in cancer‐associated fibroblasts (CAFs) affects their action on macrophages. (A) When indicated as ‘co‐culture’, CAF either wild‐type (WT) or depleted in Snail1 (KO) were incubated with bone‐marrow‐derived macrophages (BMDMΦ) for 24 h; Tomato‐labelled human MCF7 or murine AT3 mammary gland tumor cells were added and the number of Tomato‐labelled cells was determined 48 h later. When indicated ‘conditioned medium’, the conditioned medium (CM) from 24 h serum‐starved CAFs was added instead of co‐culturing the cells. Cytotoxicity was calculated as the decrease in the number of labelled cells in the presence of macrophages. The figures show the average ± SD of three experiments. (B), Macrophages were stimulated with CAFs CM for 24 h. Then, Tomato‐labelled MCF7 or AT3 cells were added and after 24 h, CD45‐positive cells were isolated by FACS and Tomato fluorescence was assessed. In (C) macrophages, either not‐stimulated or supplemented with WT or Snail1 KO CAFs CM, were incubated with GFP‐labelled beads. Presence of GFP in CD45‐positive cells was determined after 30 min. (D) BMDMΦ were stimulated with conditioned medium from CAFs for 24 h. Then, macrophages were subcutaneously co‐inoculated with AT3 cells on the flanks of NSG mice. After 3 weeks, animals were euthanized and tumors were collected and measured. The figures show the average ± SD of three experiments. Statistical significance was obtained using Student's *t*‐test; ns, not significant; *, *P* < 0.05; **, *P* < 0.01; ***, *P* < 0.001.

We assessed the impact of CAF activation on macrophage polarization in *in vitro* assays using naïve BMDMΦs and CAFs. BMDMΦs were cultured with murine WT or Snail1‐depleted (KO) CAFs (Fig. S3A), and their cytotoxic activity toward MCF7 breast tumor cells was assessed. Macrophages cultured with KO CAFs were more cytotoxic than those cultured with WT CAFs (Fig. 3A). Similar results were obtained when BMDMΦs were supplemented with the conditioned medium (CM) from either WT or KO CAFs (Fig. 3A and Fig. S3B). None of the two CM affected the total number of these cells (Fig. S3C). Similar differences in the cytotoxicity of BMDMΦ were observed when they were assayed on other tumor cells, such as murine AT3 (Fig. 3A), when macrophages were incubated with tumor cells and CAFs simultaneously (Fig. S3D,E), or when BMDMΦs were stimulated by WT or Snail1 KO MEFs (Fig. S3F).

The phagocytic activity of macrophages towards labelled tumor cells was also repressed by WT CAFs to a much higher extent than by KO CAFs, when we analyzed them against MCF7 and AT3 cells (Fig. 3B). However, both types of CAFs CM increased similarly the phagocytic activity of BMDMΦs toward fluorescent beads (Fig. 3C).

We also assessed the role of CAF‐polarized BMDMΦs on tumor formation by AT3 cells. These cells were co‐xenografted with BMDMΦs preincubated with CAF CM. Macrophages polarized with WT CAF CM increased the size of the AT3 tumors, whereas those preincubated with CM from KO CAF did not promote any significant effect (Fig. 3D), indicating that both type of fibroblasts differently affect the macrophage influence on tumor growth.

### 3.3 The gene expression pattern of active CAF‐stimulated macrophages differs from that detected in IL4‐activated macrophages

**Fig. 4:**
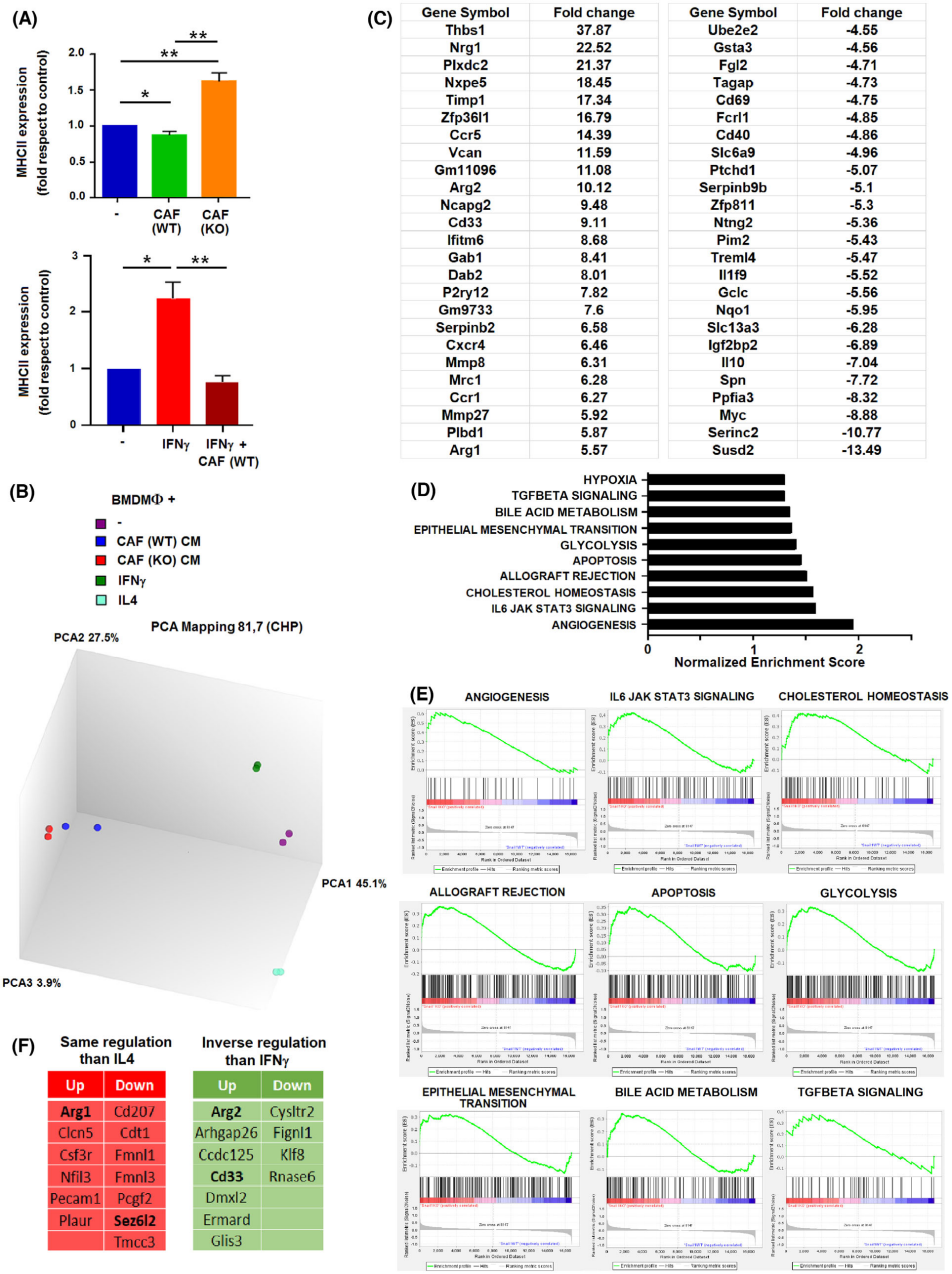
Differential gene expression in macrophages polarized with conditioned medium (CM) from wild‐type (WT) or Snail1‐depleted (KO) cancer‐associated fibroblasts (CAFs). (A) Expression of MHCII in the membrane was determined by FACS in macrophages incubated for 24 h with CM from the indicated CAFs, with IFNγ (100 units·mL^−1^) or both. The figure shows the average ± SD of three experiments. Statistical significance was obtained using Student's *t*‐test; *, *P* < 0.05, **, *P* < 0.01. (B) Diagram of the PC analysis of the genes expressed by not‐stimulated bone‐marrow‐derived macrophages or activated with IFNγ (100 units·mL^−1^), IL4 (10 ng·mL^−1^), or CM from wild‐type (WT) or Snai1‐depleted (KO) CAFs. (C) List of genes exhibiting higher differences in expression in macrophages stimulated with WT or Snail1 KO CAFs CM, either up‐regulated (left), or downregulated (right). (D, E) GSEA analysis of the categories corresponding to the genes preferentially expressed in WT versus KO CAF‐activated macrophages. (F) list of genes up‐ or downregulated in WT versus KO CAF‐activated macrophages that show a similar regulation than IL4 or an inverse regulation than IFNγ.

Before studying gene expression genes in CAF‐stimulated macrophages, we determined the expression of MHCII in the plasma membrane, since this is the parameter previously used to classify macrophages *in vivo* (see Fig. 1 and Fig. S2). BMDMΦs cultured with WT CAF CM exhibited lower membrane MHCII than those stimulated by KO CAF CM (Fig. 4A, upper panel). However, these lower levels were not accompanied with a different expression of *H2Aa*, *H2Ab*, *H2Dmb1*, or *Ciita* RNAs (Fig. S4A). As expected, IFNγ increased membrane MHCII levels, an effect that was prevented with the simultaneous incubation with CAF CM (Fig. 4A, lower panel). Moreover, CAF CM partially prevented the IFNγ‐induced increase in MHCII‐related genes (Fig. S4B), suggesting that it interferes with the canonical IFNγ polarization.

We carried out an extensive RNA analysis to compare the gene expression pattern of BMDMΦs stimulated with WT CAF CM or KO CAF CM and also with BMDMΦs polarized by IL4 or IFNγ. Principal component analysis revealed that WT CAF‐ or KO CAF‐stimulated BMDMΦs presented more similarities between themselves than with the IL4‐ or IFNγ‐induced macrophages (Fig. 4B). A list of the genes showing the highest differences between BMDMΦs polarized with WT or KO CAFs is shown in Fig. 4C. The complete list was deposited in GEO repository (see Sections 2 and 2.10).

The genes differently activated in BMDMsΦ by WT or KO CAFs belong to several categories, including angiogenesis and JAK/STAT signaling, EMT, or TGFβ signaling (Fig. 4D,E). An inspection of the genes with a higher variation revealed that some were regulated during IL4‐promoted differentiation; thus, WT CAF‐induced BMDMΦs presented a higher expression of *Arg1*, a gene upregulated by IL4, than KO CAF‐induced BMDMΦs. *Sez6l2*, a gene downregulated by IL4, was decreased in WT CAF with respect to KO CAF macrophages. A list of these genes, which we termed ‘same regulation than IL4’, is presented in Fig. 4F. Moreover, we observed that other genes repressed by IFNγ in BMDMΦs were activated by WT CAFs vs KO CAFs CM, including *Arg2* or *Cd33*; we termed these genes ‘inverse regulation than IFNγ’ (Fig. 4F).

**Fig. 5:**
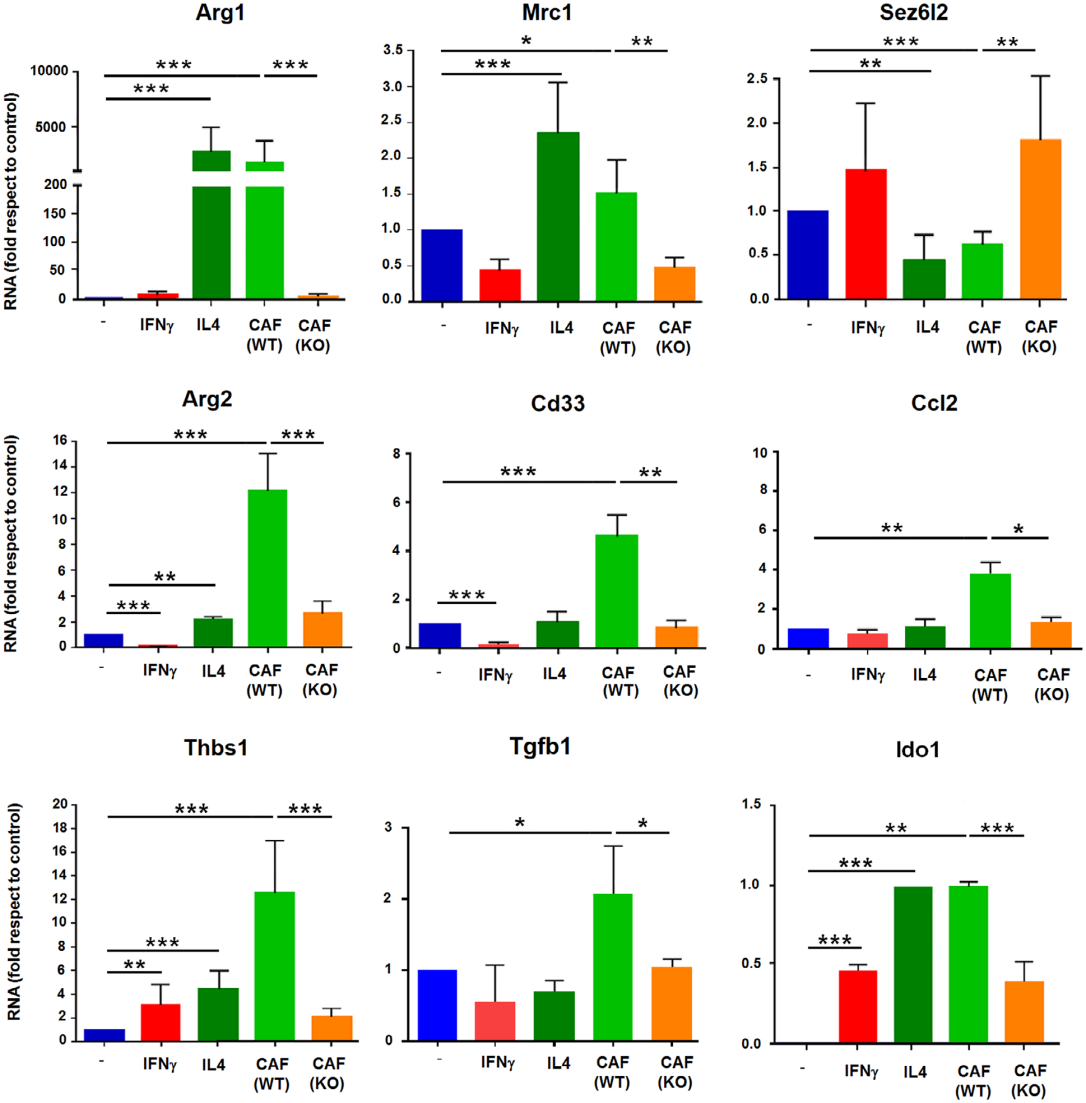
Genes specifically regulated by active cancer‐associated fibroblasts (CAFs) in macrophages do not correspond to an IL4‐induced polarization. RNA was obtained from macrophages either not stimulated, treated with conditioned medium (CM) from wild‐type (WT) or Snail1‐depleted (KO) CAFs, IFNγ (100 units·mL^−1^) or IL4 (10 ng·mL^−1^) for 24 h and analyzed by real‐time quantitative PCR coupled to retrotranscription. The figure shows the average ± SD of three experiments. Statistical significance was obtained using Student's *t*‐test; *, *P* < 0.05; **, *P* < 0.01; ***, *P* < 0.001.

We validated the expression of a set of these genes. *Arg1* and *Mrc1*, were upregulated by IL4 and WT CAF CM, but not by KO CAF CM; conversely, *Sez6l2* levels were decreased in BMDMΦs treated with either IL4 or WT CAF CM but not in those treated with KO CAF CM (Fig. 5). However, other genes stimulated by IL4, such as *Myc* or *Itgax*, were not differently increased by WT‐CAF or KO CAF CM (Fig. S4C). Moreover, *Arg2* and *Cd33* two genes downregulated by IFNγ were increased by WT CAF CM and not by KO CAF CM (Fig. 5). Macrophage expression of *Nos2*, *Ccr5*, and *Cd86*, three IFNγ‐stimulated genes, was also assessed. *Nos2* was not stimulated significantly different by WT CAF or KO CAF CM, whereas *Ccr5* was downregulated only in KO CAF; in contrast, *Cd86* was increased by WT CAF CM but not by KO CAF CM (Fig. S4C). Other genes stimulated differently by WT CAF CM versus KO CAF CM included *Ccl2*, *Thbs1*, *Tgfb1*, and *Ido1*; these genes were either not sensitive to IL4 or IFNγ, or increased similarly by both factors (Fig. 5). Therefore, the genes differently regulated in macrophages by active versus inactive CAF do not correspond to those induced by IL4 during an *in vitro* alternative polarization since they englobe some genes activated similarly than IL4 (as *Arg1*); other, inversely to IFNγ (*Arg2*); a third subset, not regulated during the canonical macrophage polarizations (*Ccl2*) and even some genes activated by IFNγ (*Cd86*).

We analyzed the expression of one of these macrophage markers, Mrc1 (CD206), in PyMT tumors. As shown in Fig. S5, macrophages expressing this protein localized close to areas with presence of activated CAFs, determined these by the expression of the CAF marker Vimentin.

The differential stimulation of *Arg1* and *Arg2* gene expression in BMDMΦ by CAF was corroborated by western blot (Fig. S6A). In WT CAF‐stimulated BMDMΦ, this upregulation was accompanied with a higher production of spermidine, a polyamine synthetized as result of the activity of Arginase (Fig. S6B). Spermidine stimulation was sensitive to the addition of difluoromethylornithine (DFMO), an inhibitor of ornithine decarboxylase [24]. Addition of this inhibitor to WT CAF‐polarized macrophages did not affect their viability (Fig. S6C) and only slightly increased their cytotoxicity (Fig. S6D), suggesting that spermidine generation is not relevant for the CAF‐imposed macrophage polarization.

The CAF stimulation on macrophage polarization was only partially mimicked by tumor cells, even if these expressed Snail1. We used BTE136 and AT3 cells, both derived from PyMT tumors but that exhibit a different expression of mesenchymal markers (Fig. S7A). CM from BTE136, a cell line with high expression of Snail1 and other mesenchymal genes, produced a small but significant decrease in the cytotoxic activity of BMDMΦs, in contrast with the lack of effect of CM from AT3 cells (Fig. S7B). Furthermore, compared with AT3 CM, the BTE136 CM only stimulated to a higher extent *Arg2* and *Thbs1* of a set of genes selected from those studied above (Fig. S7C). Lower effects were obtained with HT29 M6 tumor cells expressing Snail1 that did not decrease macrophage cytotoxicity and only stimulated *Arg1* (Fig. S7A–C).

### 3.4 Activation of the CAF‐dependent signature in macrophages is a consequence of the secretion of multiple factors

**Fig. 6:**
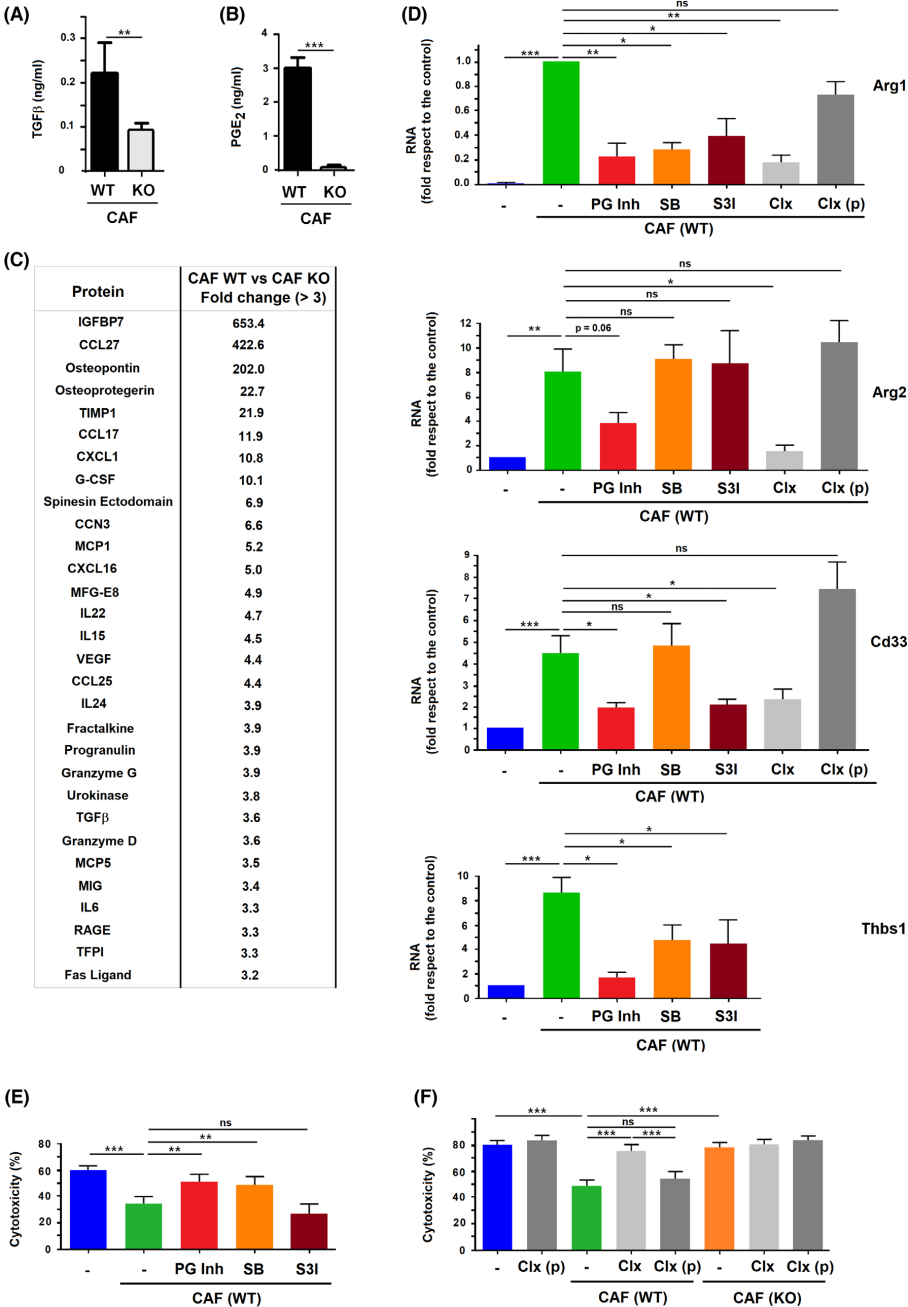
Factors specifically secreted by active cancer‐associated fibroblasts (CAFs) differentially regulate gene expression in macrophages. (A, B) Levels of TGFβ and PGE_2_ were determined in the conditioned medium (CM) from wild‐type (WT) or Snail1‐depleted (KO) CAFs. (C) Factors differently secreted by WT and KO CAFs were determined analyzing the Mouse L308 Array with the CM from both cells. A list of the 30 genes exhibiting the highest stimulation in WT CAF is shown. (D) RNA was obtained from macrophages stimulated with CM from CAF (WT) and in the presence of PGE_2_ receptor inhibitors L161 and PF (PG inh), TGFβ receptor inhibitor SB or Stat3 phosphorylation inhibitor S3I‐201 (S3I). When the effect of celecoxib (Clx) was assayed, this compound was previously added to the CAFs for 24 h (Clx, light gray bars) or, as control, just when the conditioned medium was collected (Clx(p), dark gray bars). Expression of the indicated genes was assessed by real‐time quantitative PCR coupled to retrotranscription. (E, F) Cytotoxic activity of macrophages supplemented with CM from WT CAFs and the indicated inhibitors was determined as above. The figure shows the average ± SD of three experiments. In (A, B) and (D–F) statistical significance was obtained using Student's *t*‐test; ns, not significant; *, *P* < 0.05; **, *P* < 0.01; ***, *P* < 0.001.

We also investigated the CAF‐derived factors responsible for the different gene activation. Snail1‐dependent fibroblast activation promotes a higher synthesis and production of PGE_2_ and TGFβ [4, 8]. Accordingly, WT CAF CM presented higher levels of TGFβ than KO CAF CM (Fig. 6A) and promoted a greater stimulation of Smad2 phosphorylation when added to NMuMG cells (Fig. S8A), indicating an elevated secretion of active TGFβ. Similar results were observed when Smad2 phosphorylation was assessed in CAF‐treated BMDMΦs (Fig. S8B). These results also agree with the observation that ‘TGFβ signaling genes’ was one of the top categories differently expressed in macrophages stimulated by WT CAF versus KO CAF (see Fig. 4D,E). PGE_2_ levels were also considerably higher in WT CAF CM than in KO CAF CM (Fig. 6B). We also analyzed the secretome of these CAFs. The release of a considerable number of cytokines and growth factors was increased in active (WT) versus inactive (Snail1 KO) CAFs; only few factors were more abundant in KO CAF CM (Fig. 6C and Table S3). Another category differently expressed in WT CAF‐activated versus KO CAF‐activated macrophages was IL6/JAK/STAT3 signaling; accordingly, IL6 and other cytokines were elevated in WT CAF CM (Fig. 6C). This medium increased Stat3 phosphorylation in BMDMΦs to a higher extent than KO CAF CM (Fig. S8C,D). Stat3 phosphorylation in BMDMΦs was inhibited with S3I‐201 (S3I), a Stat3 antagonist, and also with two inhibitors of PGE_2_ receptors, EP2 and EP4 (Fig. S8C,D).

We analyzed the effect of these secreted factors on the expression in macrophages of CAF‐stimulated genes. PGE_2_ and TGFβ potently activated the expression of *Arg1* and *Arg2* genes in contrast to osteopontin (OPN) or IL6 (Fig. S8E); these stimulations were sensitive to the addition of L161982 and PF04418948, antagonists of PGE_2_ receptors EP2 and EP4, respectively, as well as to the TGFβ Receptor inhibitor SB505125 (SB). On the contrary, OPN and IL6 stimulated *Cd33* to a higher extent than PGE_2_ or TGFβ (Fig. S8E). PGE_2_ and TGFβ, but not OPN, also significantly decreased the cytotoxic activity of macrophages (Fig. S8F).

We examined the effect of inhibitors of these factors on the expression in BMDMΦ of CAF‐stimulated genes. Of note, CAF‐stimulated genes showed a different sensitivity to PGE_2_, TGFβ, and Stat3 inhibitors: PGE_2_ antagonists were the most potent and decreased the four genes studied whereas inhibitors of TGFβ receptor or Stat3 were more selective (Fig. 6D and Fig. S9). The effect of PGE_2_ inhibitors on *Arg1*, *Arg2*, *Cd33*, and *Sez6l2* gene expression in macrophages was also confirmed by the pretreatment of CAFs with celecoxib (Fig. 6D and Fig. S9), an inhibitor of prostaglandin production.

We also assessed the action of these compounds on the attenuation by CAFs of the macrophage cytotoxic activity. TGFβ and PGE_2_ inhibitors significantly rescued the decrease in the cytotoxic activity caused by incubation of BMDMΦ with WT CAF (Fig. 6E,F).

These results indicate that activation of gene expression in macrophages by CAFs is a consequence of the secretion of multiple cytokines and growth factors, being the increased expression of PGE_2_ and TGFβ relevant for the modification of the macrophage function.

### 3.5 CAF‐polarized macrophages activate other tumor stromal cells

**Fig. 7:**
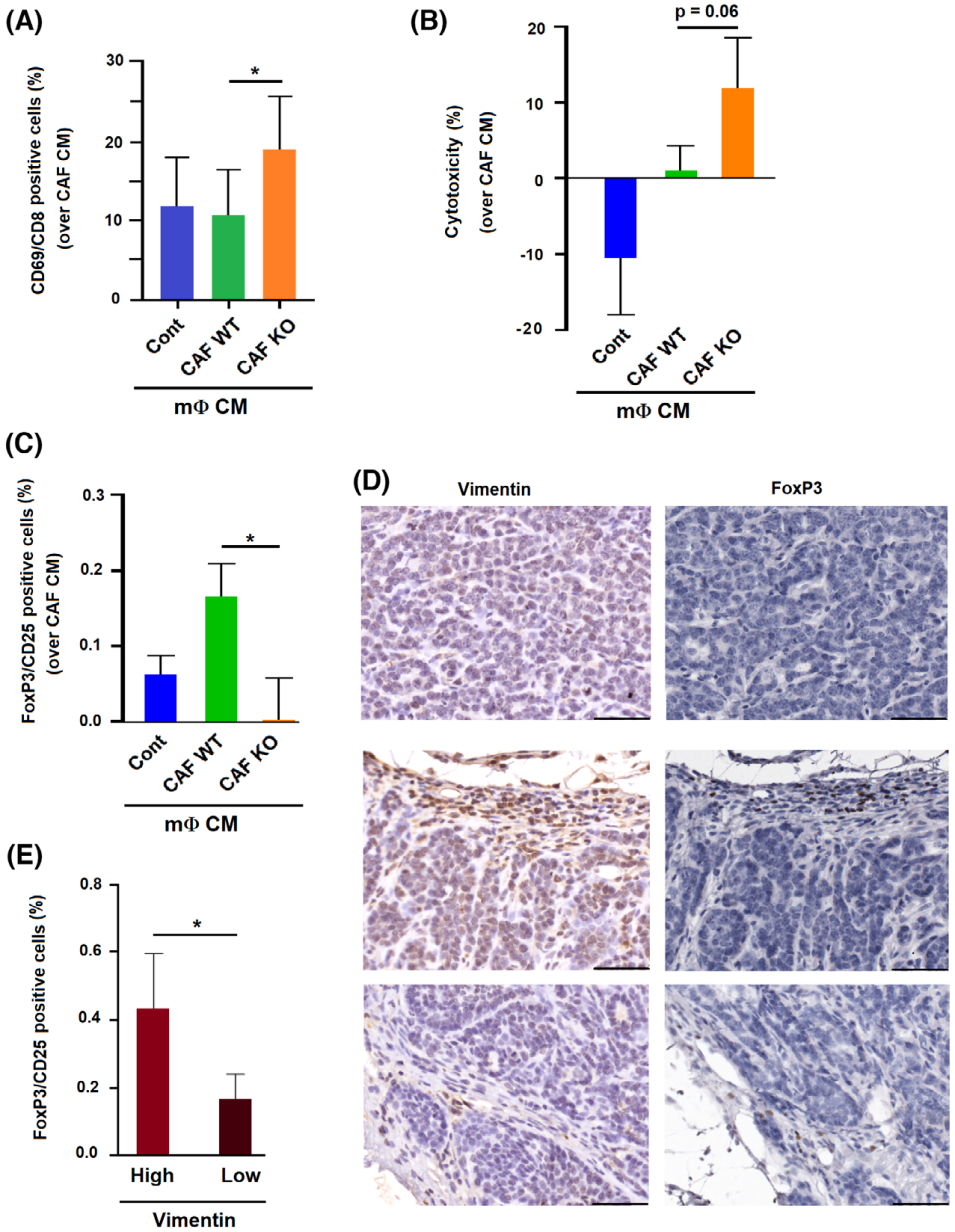
Functional effects of cancer‐associated fibroblast (CAF)‐induced macrophage polarization on lymphocytes. (A–C) Macrophage conditioned medium (CM) was used to stimulate lymphocytes; activation of CD8 lymphocytes (A) or regulatory T cells (T‐reg) (C) was determined by assessing the number of CD69/CD8‐positive or FoxP3/CD25‐positive cells by flow cytometry. Cytotoxicity of CD8 cells on OVA‐AT3 cells was also determined. (B) The average ± SD of three experiments is shown. (D, E) Expression of CAF and T‐reg markers vimentin and FoxP3, respectively, was determined in four PyMT tumors as indicated in Section 2. In (D) representative images of areas with low (upper panels) and high (middle and lower panels) vimentin expression with the corresponding staining of FoxP3 in the same areas. The bar corresponds to 50 μm. In (E) a quantification of the number of FoxP3 positive cells in areas with low (*n* = 8) or high (*n* = 8) vimentin expression. In (A–C and E) statistical significance was obtained using Student's *t*‐test; *, *P* < 0.05.

We analyzed the possible actions of CAF‐polarized BMDMΦ on other cells in the TME. Although not significant, we detected a lower activation of HMEC1 endothelial cell invasion through Matrigel when these cells were cultured with WT CAF‐polarized BMDMΦ CM compared with KO CAF‐polarized cells (Fig. S10A). This is probably related to the higher expression of the pro‐angiogenic factors VEGFα and FGF2 by active CAF‐polarized macrophages (Fig. S10B). We also determined the different abilities of BMDMΦs to activate other immune cells. The number of active CD8^+^ lymphocytes increased in the presence of CM of BMDMΦs treated with KO CAF CM but not of those treated with WT CAF CM (Fig. 7A); accordingly, CD8‐dependent toxicity over tumor cells was also higher, although the results were not significant (Fig. 7B). Of note, the effect of the remnant CAF CM was subtracted in all these assays. Since tumor‐associated macrophages modulate immunosuppressive T‐regs [25], we also analyzed these cells. CM from BMDMΦs polarized with WT CAF increased the number of active T‐regs to a higher extent than the corresponding control (Fig. 7C). Moreover, T‐reg presence in PyMT tumors associated with the expression of CAF marker Vimentin (Fig. 7D,E).

### 3.6 An alternative macrophage signature associates with CAF activation in human breast tumors

**Fig. 8:**
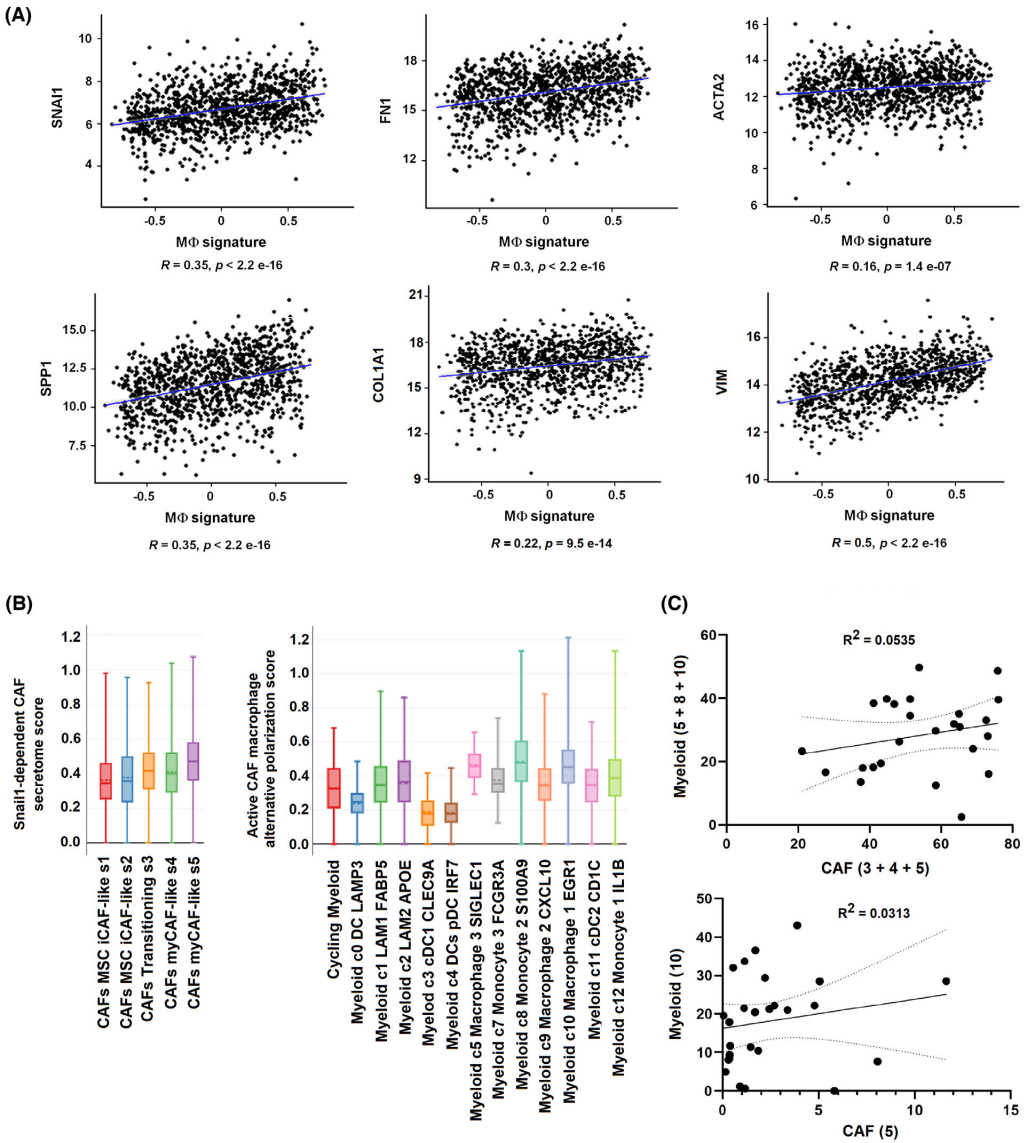
Macrophage gene signature specific for cancer‐associated fibroblast (CAF) activation correlates with mesenchymal gene expression in human tumors. (A) A signature characteristic of CAF‐activated macrophages was analyzed using the GSVA method in TCGA Breast Invasive Carcinoma PanCancer Atlas dataset; Spearman's correlation analysis showed positive associations among this macrophage's signature (*PLXDC2/TIMP1/CCR5/ARG2/CD33/CXCR4/MRC1/CCR1/ARG1*) and markers of active fibroblasts (*SNAI1*, *FN1*, *ACTA2*, *SPP1*, *COL1A1*, and *VIM*). The blue line indicated the regression of the values. (B) Breast cancer patients’ single‐cell RNA‐Seq data [22] were obtained and analyzed online at https://singlecell.broadinstitute.org/single_cell/portal as indicated in Section 2. CAF populations were scored by their expression of CAF WT versus CAF KO‐secreted proteins; myeloid cells, by the expression of genes up‐regulated in CAF‐activated macrophages. Each box is defined between Q1 and Q3 values and includes a dashed line (median) and a solid line (average). The bars represent the maximum and the minimum value of the series. The association between CAF and myeloid subpopulations in the cohort of 26 breast tumors is also shown (C). Statistical significance was assessed with the goodness‐of‐fit test.

From the genes with a differential expression in BMDM stimulated by active versus *Snail1* KO CAF CM, we selected nine that were upregulated and showed a preferential expression in macrophages (*Plxdc2*, *Timp1*, *Ccr5*, *Arg2*, *Cd33*, *Cxcr4*, *Mrc1*, *Ccr1*, and *Arg1*). We interrogated public databases for the association of this signature with a high mesenchymal infiltration in breast tumors, defined by those that exhibited a high expression of CAF markers. As shown in Fig. 8A, in these human neoplasms, the macrophage signature correlated with the expression of *SNA1*, *FN1*, *ACTA2* (αSMA), *SPP1* (osteopontin), *COL1A1*, and *VIM*, all genes that are specific for active CAFs, indicating that this gene signature is associated with CAF activation. This macrophage alternative gene signature was also related to a higher endothelial infiltration, defined by *PECAM* expression (Fig. S11A) and also with that of *FGF2* but not with *VEGFA*. Finally, the CAF‐activated macrophage signature also correlated with the presence of T‐reg cells, as determined by *FOXP3* and *IKZF2* expression (Fig. S11B).

We also analyzed recently published single‐cell RNA‐Seq data from a cohort of 26 breast cancer patients [22]. First, we scored the CAFs subpopulations obtained in this study depending on the expression of the secreted proteins described in Fig. 6C for our WT CAFs. The CAFs subpopulations *Transitioning s3*, *myCAF‐like s4*, and *myCAF‐like s5* showed a higher level of these factors compared with other subpopulations, being *myCAF‐like s5* the subpopulation with the highest score (Fig. 8B). This CAF subpopulation also ranked the highest when it was analyzed considering the expression of the CAF activation markers used in Fig. 8A, all also dependent on Snail1 (Fig. S12). Furthermore, we analyzed the myeloid cell subpopulations using the mean of their expression of the 25 genes with the highest expression in the macrophage polarization induced by active versus inactive CAFs; therefore, the 25 genes showing more activation in Fig. 4C. *Myeloid c5 Macrophage 3 SIGLEC1*, *Myeloid c8 Monocyte 2 S100A9*, and *Myeloid c10 Macrophage 1 EGR1* presented the highest scores (Fig. 8B). Finally, in the cohort of 26 tumors, we found a strong positive correlation between the abundance of s3 + s4 + s5 CAFs (in percentage of total CAFs) and the presence of c5 + c8 + c10 myeloid populations (measured as percentage of total myeloid cells; Fig. 8C). A similar association was observed when we compared just CAF s5 and myeloid c10 (*Macrophage 1 EGR1*), further suggesting that CAF activation promotes the polarization of macrophages to this alternative phenotype.

## 4 Discussion

We have extensively studied the role of Snail1 in mammary gland tumorigenesis. PyMT Snail1 KO mice show a higher survival than wild‐type mice, something that has attributed to Snail1 expression in CAFs, since these cells exhibit the highest expression of this factor. Moreover, Snail1 has been demonstrated to be essential for CAF activation. Besides this effect on CAFs, the results presented in Fig. 1 show that PyMT mammary gland tumors generated in Snail1‐deficient mice display a different macrophage polarization than those generated in control mice. Although the contribution of these altered macrophages in the increased survival remains to be studied, these results indicate that Snail1 directly or indirectly controls the polarization of these cells. After demonstrating that Snail1 is not expressed in macrophages, we have studied the effect of CAF activation in macrophage polarization analyzing the functional differences observed between the polarization of naïve BMDMΦ promoted by wild‐type and Snail1‐depleted, inactive CAFs. An association between CAFs and the alternative activation of macrophages has been observed in different tumors [26, 27, 28, 29], although a clear functional implication of CAF activation in macrophage polarization has not been investigated. Our study determines that compared with Snail1‐depleted, inactive CAFs, active CAFs induce a polarization state in BMDMΦs characterized by a lower cytotoxicity. This state does not correspond to an alternative IL4‐induced polarization since besides up‐regulating several classical markers of this differentiation, such as *Arg1*, CAFs also increase other gene products that are not modified by IL4 but downregulated by the M1 inducer IFNγ, such as *Arg2*. Moreover, other genes that are not modulated by IL4 or IFNγ are also specifically activated. Of note, WT CAFs also stimulated BMDMΦs expression of *Cd86*, an archetypical M1 marker, to a higher extent than KO CAFs. This agrees with single‐cell transcriptome analyses that have detected expression of genes up‐regulated by IL4 and IFNγ in some tumor‐derived macrophages [17]. It is also remarkable that, as shown in Fig. 4, although macrophages polarized by active or inactive CAFs are functionally distinct, they present more resemblances in gene expression than to those activated with IFNγ or IL4.

Our results also show that BMDMΦs polarized with active or inactive CAFs differently phagocyte tumor cells but do not show differences in fluorescent beads phagocytosis (see Fig. 3). These results suggested us that at least part of the effect of active CAFs on macrophages is cell‐dependent, and CAFs modulate the macrophage attack on tumor cells. The activity of macrophages on tumor cells is controlled through the interaction of tumoral CD47 with its receptor in macrophages, signal regulatory protein a (SIRPα) [30]. We have not detected significant differences in the transcriptome analysis in the expression of *SIRPα* between BMDMΦ polarized with active and inactive CAFs; in contrast, the levels of *Thbs1* are considerably higher in active CAFs‐polarized BMDMΦ (see Fig. 5). Thbs1 is also a high‐affinity receptor for CD47 and has been proposed to regulate CD47‐SIRPα binding [31]; moreover, it also contributes to the inhibition of other immune cells [31]. Therefore, it is also possible that the high expression of Thbs1 in CAF‐activated BMDMΦ might participate in the inhibition of the cytostatic and phagocytic activity of these cells on tumoral cells but not in the phagocytosis of beads.

As shown in Fig. 2, neither Snail1 protein nor its RNA was detected in macrophages. Some authors have previously reported Snail1 expression in these cells by immunohistochemistry [32]. However, these results were obtained with a Snail1 polyclonal antibody not currently available that might have recognized another related protein. Snail1 has also been detected in the THP‐1 macrophage cell line when stimulated with TGFβ [33]. In contrast, we have not observed Snail1 expression in BMDMΦs after they were stimulated with the CM from CAFs, with IFNγ or IL4. Only very low amounts of Snail1 RNA were detected, less than 1% of the values observed in not‐stimulated MSCs (see Fig. 2). Other EMT transcription factors, such as *Twist1* or *Snail2*, were not expressed by BMDMΦs; only *Zeb1* was detected in these cells, at levels corresponding to 3% of non‐stimulated MSCs. Zeb1 has been reported to be expressed by F4/80 low macrophages [34]. We have not observed Snail1 expression in this macrophage subtype.

Macrophages are very plastic cells and very sensitive to changes in the local microenvironment [35]. They undergo extensive changes in gene transcription upon exposition to cytokines that generate a hostile or permissive immune response. It has been proposed that these changes are relatively transient, and that after removal of the polarizing cytokine, they can return to the baseline state [36]. Our results imply that an active CAF‐rich TME promotes the polarization of BMDMΦs to an immunosuppressive phenotype, preventing its cytotoxic activity against tumor cells and also enhancing the activation of the T‐reg immunosuppressive cells. Accordingly, an analysis of human tumors shows a close association of the expression of a gene signature characteristic of CAF‐activated macrophages with genes specific of T‐reg activation.

Snail1 depletion in CAF affects their production of TGFβ, PGE_2,_ and OPN, three molecules reported to control macrophage differentiation to the alternative M2 phenotype [37, 38, 39, 40, 41, 42]. The precise mechanisms used by Snail1 to control these factors have only partially unveiled. For instance, PGE_2_ production needs the activation of cyclooxygenase 2, an enzyme required for prostaglandin synthesis, as well as the direct repression of 15‐hydroxyprostaglandin dehydrogenase, involved in PGE_2_ degradation [8, 43]. Although Snail1 has been traditionally considered a transcriptional repressor, Snail1 activation of mesenchymal genes is associated with a direct interaction of this transcriptional factor to their promoters, although this has only been documented for some specific genes [5, 44, 45]. In our studies, PGE_2_ is the most potent factor inducing our alternative program and repressing cytotoxicity; consequently, its inhibition exerts a more complete effect than that of TGFβ (Fig. 6 and Fig. S4). Moreover, other cytokines that are also upregulated in WT CAF versus CAF Snail1 KO cells, such as IL6 and GM‐CSF, have also been implicated in the alternative differentiation of macrophages [37, 46]. At this regard, OPN is a poorer activator than PGE_2_ or TGFβ of the alternative, CAF‐induced polarization (see Fig. S4), as assessed by the expression of *Arg1* or *Mrc1*. In any case, we cannot discard that other factors secreted by CAFs, such as those indicated in [29], may also play a role.

Our results also show that Snail1 expression in tumor cells is much less efficient than CAFs in promoting macrophage differentiation toward the alternative phenotype. Other authors have shown that Snail1 in tumor cells enhances macrophage recruitment [6] and their alternative M2 polarization [47, 48]. In our MMTV‐PyMT cancer model, the number of total macrophages in tumors was not different in WT or Snail1‐depleted mice, suggesting that other tumor cells are more relevant than active CAFs in attracting macrophages. Moreover, the differences in the activation of IL4‐induced genes were much less relevant when macrophages were activated with the CM from tumor cells transfected with Snail1 than with CAF CM, probably reflecting that Snail1 only produces a partial EMT in tumor cells or that it cannot promote the secretion of PGE_2_. It is possible that the differences observed by other authors when comparing Snail1‐deficient versus WT tumor cells on macrophage differentiation is due to the higher capability of Snail1‐expressing cells to activate CAF [49]. In any case, the fact that macrophages also secrete factors that activate CAFs [28, 50] indicates that tumor cells, CAFs, and macrophages participate in a complex network of activation, likely involved in the generation and progression of human tumors.

## 5 Conclusions

Our results show that active, Snail1‐expressing cancer‐associated fibroblasts promote the polarization of macrophages toward a phenotype with low cytotoxicity that also enhances the activation of T‐reg cells. With respect to inactive CAF‐treated cells, these CAF‐activated macrophages present an alternative polarization that does not correspond to a classic IL4‐induced phenotype and is characterized by the expression of genes that are (a) normally induced in the IL4‐promoted macrophage polarization, (b) downregulated by IFNγ, or (c) not altered during these two canonical differentiations. This CAF effect is dependent on their secretion of PGE_2_ and TGFβ, since inhibitors of these factors prevent this alternative macrophage polarization.

## Conflict of interest

The authors declare no conflict of interest.

## Author contributions

RM and AGdH conceived and designed the study. MB‐O, RO‐S, BdV‐P, PM‐G, and RM performed the experimental work. AB analyzed the polyamines. MB‐O and RP did the bioinformatic analysis. RP helped with the tumor analysis. AGdH, with contributions from MB‐O and RO‐S, wrote the manuscript. All authors read and approved the final manuscript.

### Peer review

The peer review history for this article is available at https://www.webofscience.com/api/gateway/wos/peer‐review/10.1002/1878‐0261.13454.

## Supporting information


**Fig. S1.** Snail1 staining and morphology of MMTV tumors.
**Fig. S2.** FACS gating strategy to identify macrophages and their polarization status.
**Fig. S3.** Activation of mesenchymal cells alters the cytotoxic activity of macrophages.
**Fig. S4.** Gene expression in macrophages polarized with conditioned medium from wild‐type or Snail1‐depleted cancer‐associated fibroblasts.
**Fig. S5.** Expression of cancer‐associated fibroblasts (CAFs)‐dependent bone‐marrow macrophage activation marker Mrc1 and active CAF protein vimentin associated in PyMT.
**Fig. S6.** Macrophages polarized with conditioned medium from active cancer‐associated fibroblasts (CAFs) present an elevated Arg1 and 2 expression.
**Fig. S7.** Snail1‐expressing tumor cells are less effective than cancer‐associated fibroblasts (CAFs) in the promotion of the alternative macrophage polarization.
**Fig. S8.** Prostaglandin E_2_ (PGE_2_) stimulates Arg1 and Arg2 gene expression and represses macrophage cytotoxicity.
**Fig. S9.** Prostaglandin E_2_ (PGE_2_) and TGFβ receptor inhibitors reverse the repression of *Sez6l2* by cancer‐associated fibroblasts (CAF)‐stimulated macrophages.
**Fig. S10.** Functional effects of cancer‐associated fibroblasts (CAFs)‐induced macrophage polarization on endothelial cells.
**Fig. S11.** Macrophage gene signature specific for cancer‐associated fibroblasts (CAF) activation correlates with endothelial cell markers and genes characteristic of activated regulatory T (T‐regs) cells.
**Fig. S12.** Cancer‐associated fibroblast (CAF) *myCAF‐like s5* signature presents the highest association with CAF activation markers.
**Table S1.** Antibodies used in this article.
**Table S2.** Primers used in the real‐time quantitative PCR coupled to retrotranscription.
**Table S3.** Secreted factors differently expressed in wild‐type versus Snail1‐depleted cancer‐associated fibroblasts.

## Data Availability

The data that support the findings of this study are available from the corresponding author (agarcia@imim.es) upon reasonable request. The data that support the findings of the microarray analysis mentioned in 2.10 have been deposited in NCBI's GEO (GSE206404, token sbsxciochrmpnon).
