# Supplementary material for: Analyzing the role of cancer‐associated fibroblast activation on macrophage polarization
Source: Mol Oncol. 2023 Jun 7;17(8):1492–513. doi: 10.1002/1878-0261.13454 (PMC10399715; doi:10.1002/1878-0261.13454)
Supplement: Supplementary file 1 — Fig. S1. Snail1 staining and morphology of MMTV tumors. Fig. S2. FACS gating strategy to identify macrophages and their polarization status. Fig. S3. Activation of mesenchymal cells alters the cytotoxic activity of macrophages. Fig. S4. Gene expression in macrophages polarized with conditioned medium from wild‐type or Snail1‐depleted cancer‐associated fibroblasts. Fig. S5. Expression of cancer‐associated fibroblasts (CAFs)‐dependent bone‐marrow macrophage activation marker Mrc1 and active CAF protein vimentin associated in PyMT. Fig. S6. Macrophages polarized with conditioned medium from active cancer‐associated fibroblasts (CAFs) present an elevated Arg1 and 2 expression. Fig. S7. Snail1‐expressing tumor cells are less effective than cancer‐associated fibroblasts (CAFs) in the promotion of the alternative macrophage polarization. Fig. S8. Prostaglandin E2 (PGE2) stimulates Arg1 and Arg2 gene expression and represses macrophage cytotoxicity. Fig. S9. Prostaglandin E2 (PGE2) and TGFβ receptor inhibitors reverse the repression of Sez6l2 by cancer‐associated fibroblasts (CAF)‐stimulated macrophages. Fig. S10. Functional effects of cancer‐associated fibroblasts (CAFs)‐induced macrophage polarization on endothelial cells. Fig. S11. Macrophage gene signature specific for cancer‐associated fibroblasts (CAF) activation correlates with endothelial cell markers and genes characteristic of activated regulatory T (T‐regs) cells. Fig. S12. Cancer‐associated fibroblast (CAF) myCAF‐like s5 signature presents the highest association with CAF activation markers. Table S1. Antibodies used in this article. Table S2. Primers used in the real‐time quantitative PCR coupled to retrotranscription. Table S3. Secreted factors differently expressed in wild‐type versus Snail1‐depleted cancer‐associated fibroblasts. [file MOL2-17-1492-s001.pdf]

Analyzing the role of cancer-associated fibroblast activation on macrophage polarization

Marina Bruch-Oms, Rubén Olivera-Salguero, Rocco Mazzolini, Beatriz del Valle-Pérez, Paula Mayo-González, Ángel Beteta, Raúl Peña, and Antonio García de Herreros

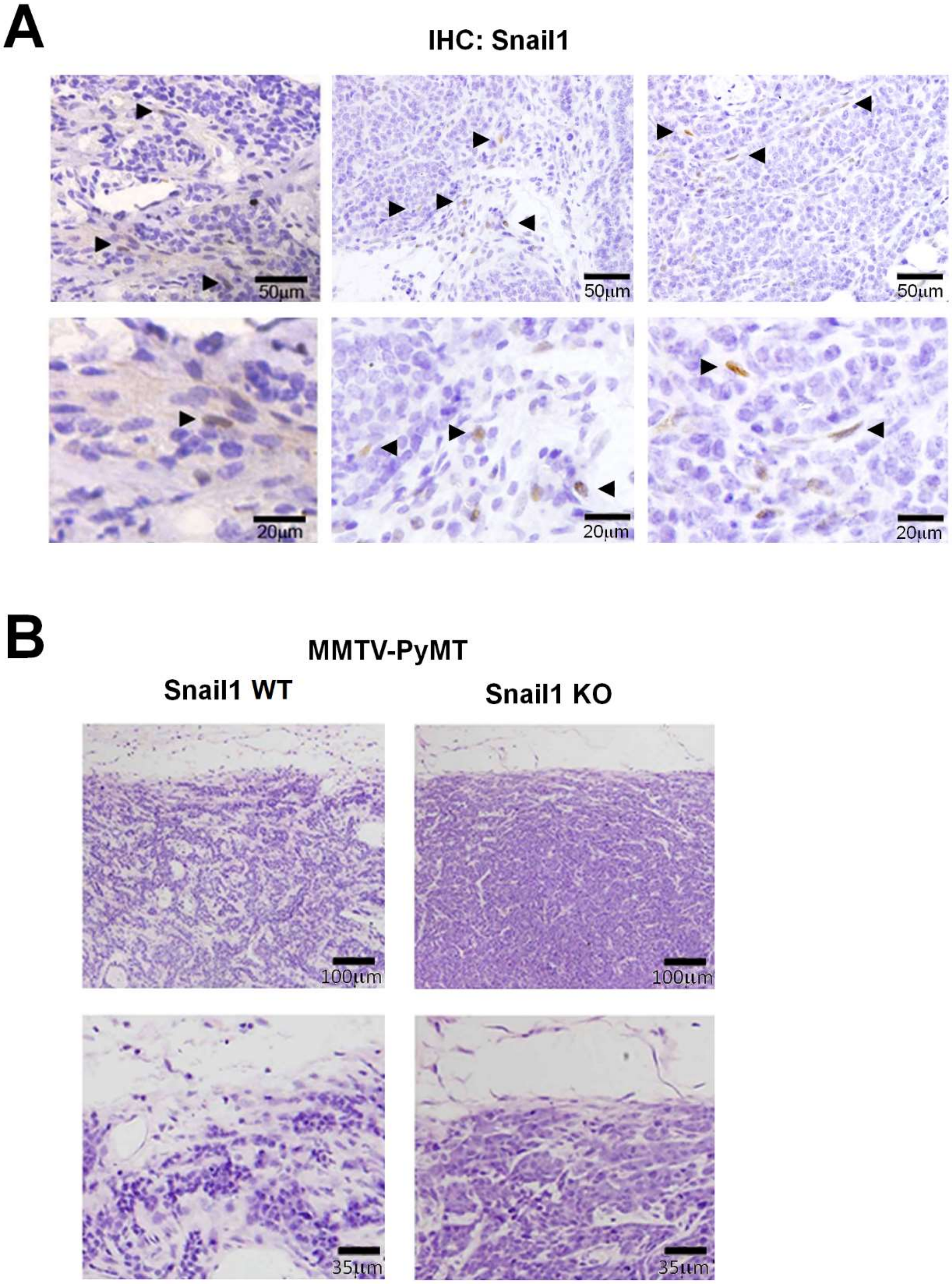

Figure S1. Snail1 staining and morphology of MMTV tumors. A, Snail1 staining of MMTV-PyMT tumors was performed as indicated in Methods. The figure presents representative images (upper row) with magnification (lower row) of Snail1-expressing cells (labelled with arrowheads). **B,** Representative images of hematoxylin-eosin staining of the invasion from of MMTV-PyMT tumors generated in wild-type (WT) or Snail1 KO mice.

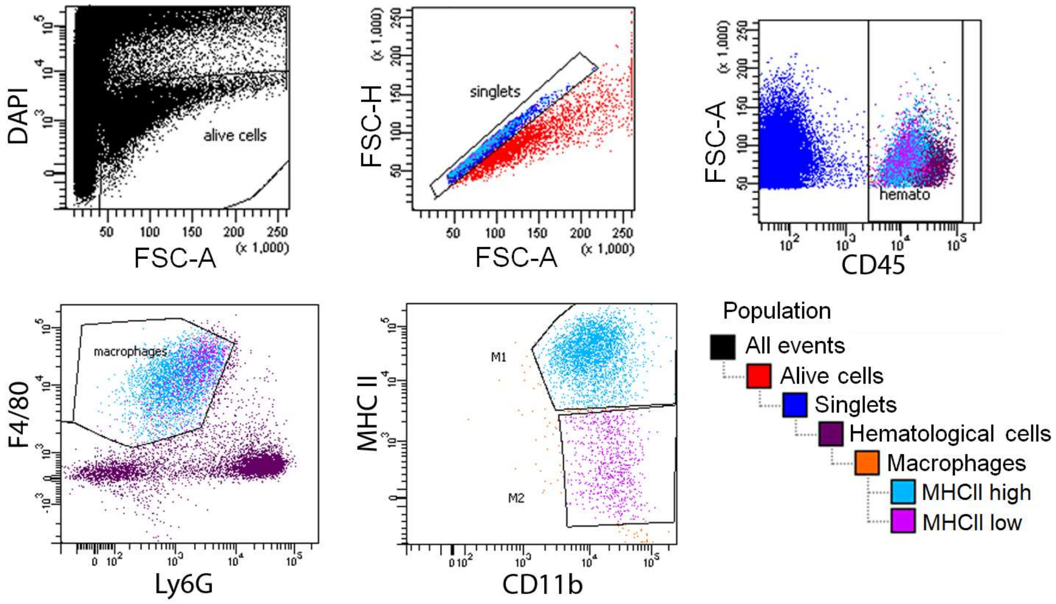

Figure S2. FACS gating strategy to identify macrophages and their polarization status. MMTV-PyMT mammary tumors were harvested and dissociated for single cell recovery. Representative plots of living CD45+, F4/80+, Ly6G-, CD11b+ and MHC-low or -high macrophages are shown.

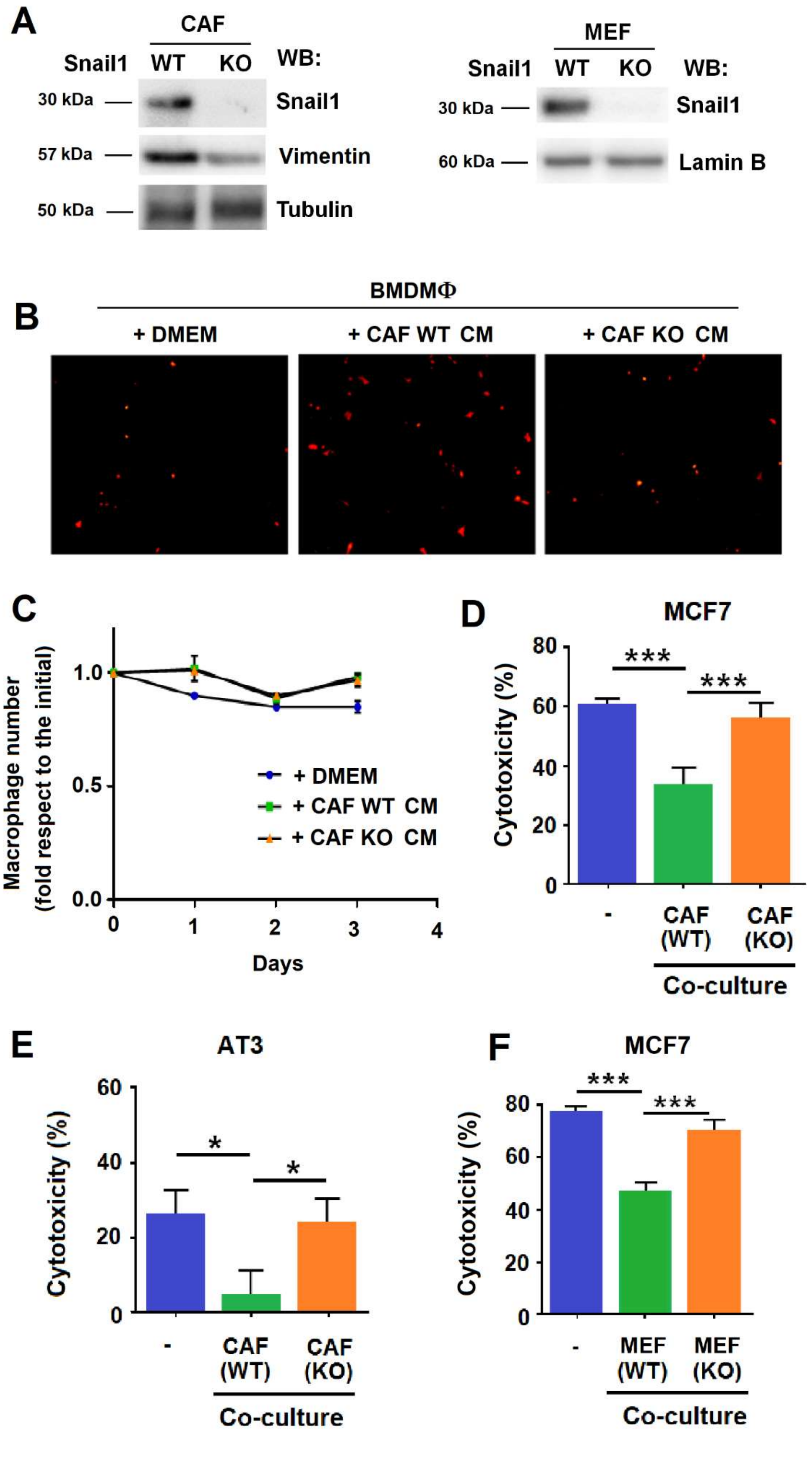

Figure S3. Activation of mesenchymal cells alters the cytotoxic activity of macrophages. A, Snail1 expression in cancer-associated fibroblasts (CAFs) or murine

embryo fibroblasts (MEFs), as assessed by western blot. **B,** Tomato-labelled human MCF7 mammary tumor cells were incubated with bone marrow-derived macrophages (BMDMΦ) and conditioned medium (CM) from the indicated CAFs. A representative micrograph showing Tomato-labelled cells 48 h later is shown. **C**, Effect of CAF CM on macrophage viability. Number of macrophages was determined by MTT assays. **D-F,** Tomato-labelled human MCF7 or murine AT3 mammary tumor cells were incubated with CAF or MEF either Snail1 WT or KO for 24 h. Bone marrow-derived macrophages were added and the number of Tomato-labelled cells was determined 48 h later. In C-F the average ± SD of three experiments is shown. Statistical significance was obtained using Student's t-test; *, $P < 0.05$; ***, $P < 0.001$.

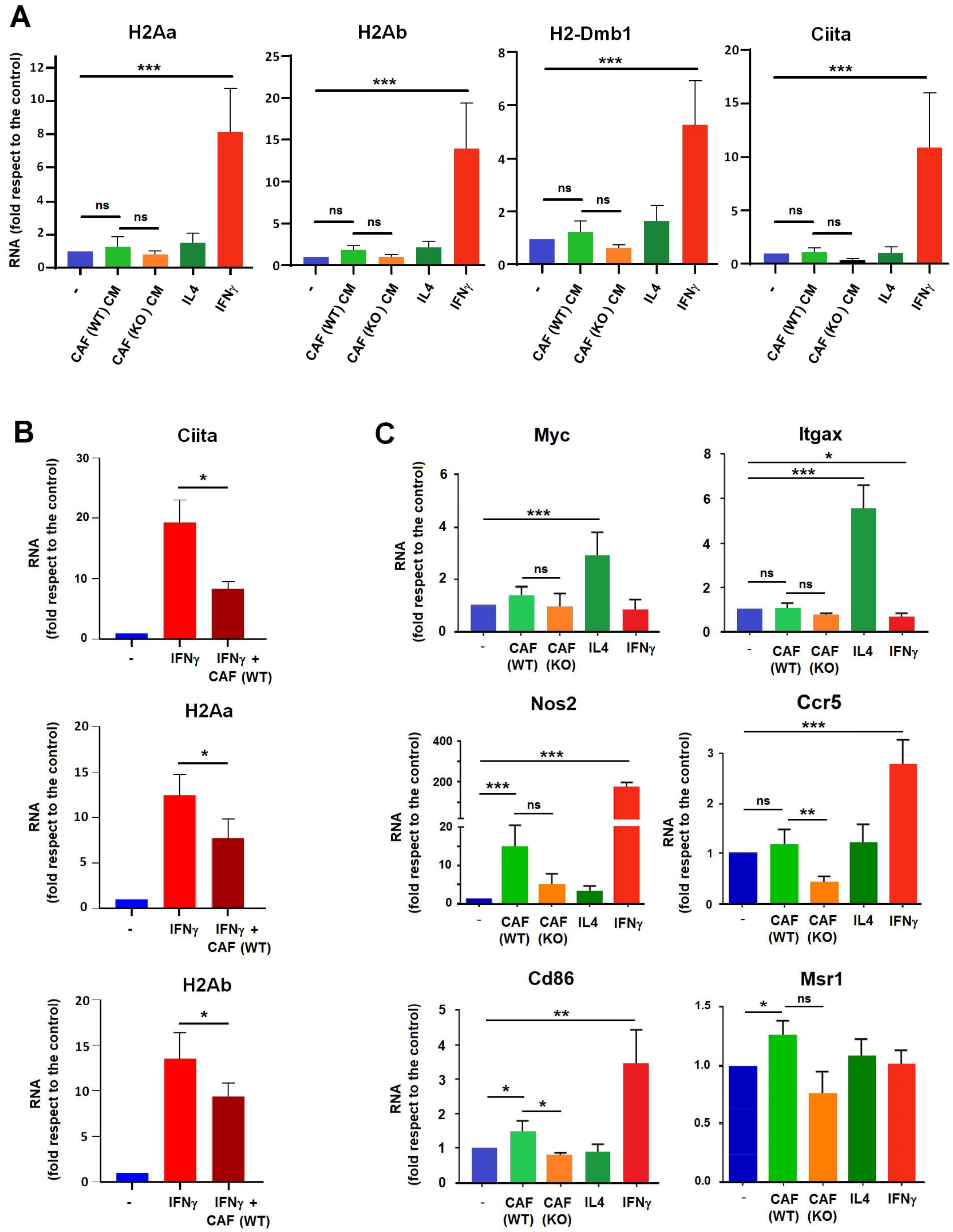

Figure S4. Gene expression in macrophages polarized with conditioned medium from wild-type or Snail1-depleted cancer-associated fibroblasts (CAFs). A-C, RNA analysis in not-stimulated macrophages or activated with conditioned medium (CM) from wild-type (WT) or Snail1-depleted (KO) CAFs, with IL4, IFNγ or IFNγ plus WT CAFs CM. All treatments were for 24 h. The figure shows the average ± SD of

three experiments. Statistical significance was obtained using Student’s t-test; ns, not significant; *, $P < 0.05$; **, $P < 0.01$; ***, $P < 0.001$.

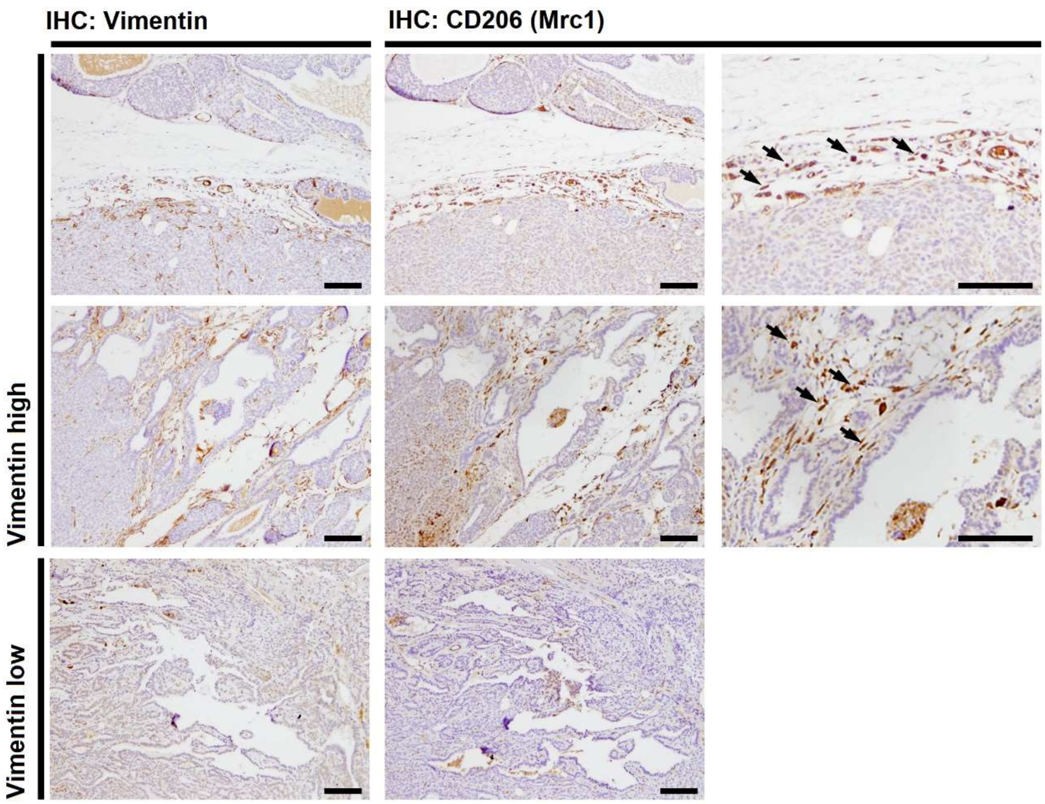

Figure S5. Expression of cancer-associated fibroblasts (CAFs)-dependent bone marrow macrophage activated marker Mrc1 and active CAF protein vimentin associate in PyMT. Representative images of areas with low and high vimentin expression with the corresponding staining of CD206 (Mrc1) in the same areas. The bar corresponds to 100 μm. The right panels correspond to magnifications of the middle panels; the arrows identify macrophages stained with the CD206 antibody.

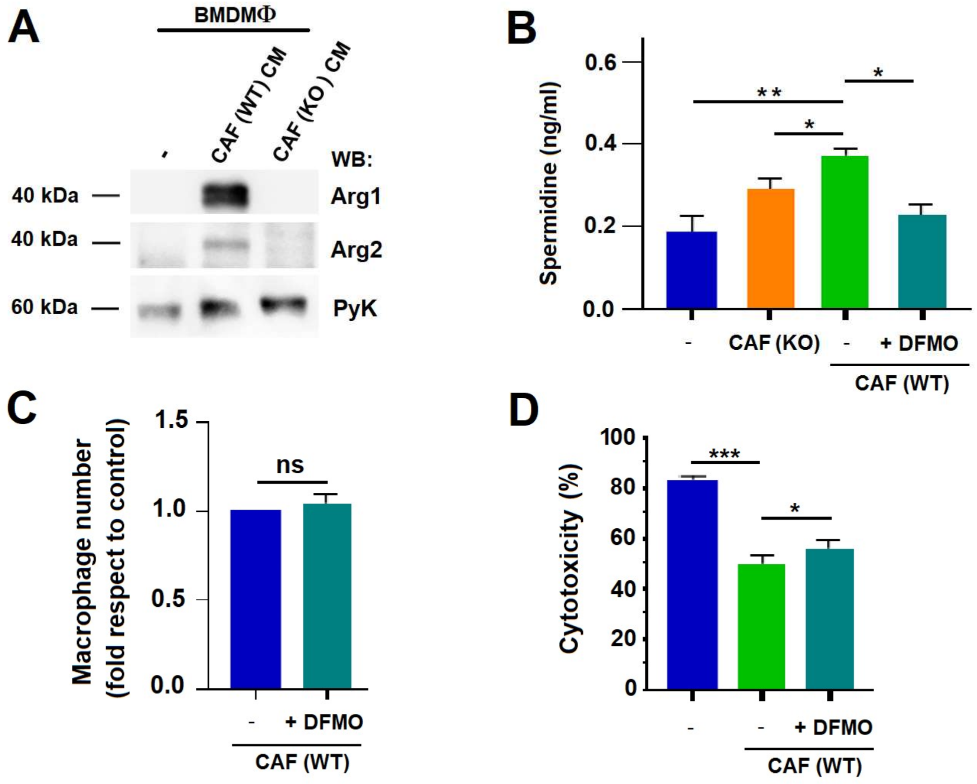

Figure S6. Macrophages polarized with conditioned medium from active cancer-associated fibroblasts (CAFs) present an elevated Arg1 and 2 expression. A, Analysis of Arg1 and 2 in total extracts of bone-marrow-derived macrophages (BMDMΦ) incubated with conditioned medium (CM) from CAFs. **B**, Spermidine levels in macrophages treated as in A and incubated with DFMO (100 μM). **C**, Effect of DFMO on macrophage viability. Number of macrophages was determined by MTT assays. **D,** Tomato-labelled human MCF7 were incubated with CAFs for 24 hours and later with BMDMΦ and the indicated inhibitors for 48 h. Macrophage-induced cytotoxicity was assessed as indicated in Methods. In B-D the average ± SD of three experiments is shown. Statistical significance was obtained using Student's t-test; ns, not significant; ns, not significant, *, P <0.05; **, P <0.01; ***, P <0.001.

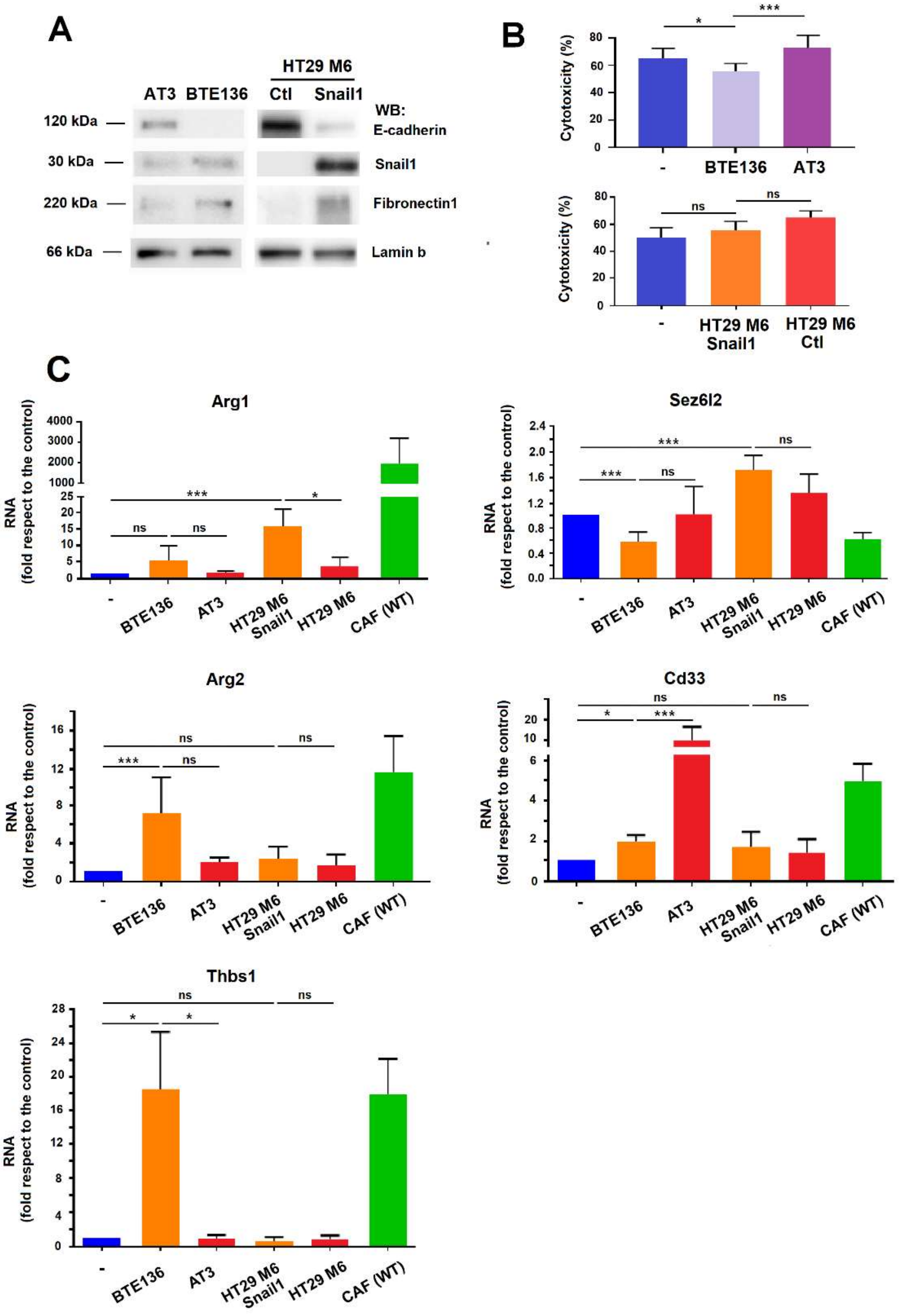

Figure S7. Snail1-exppressing tumor cells are less effective than cancer-associated fibroblasts (CAFs) in the promotion of the alternative macrophage polarization. A, Expression of Snail1 and epithelial (E-cadherin) or mesenchymal (Fibronectin) markers in breast tumor AT3 and BTE136 cells or HT29 M6 cells transfected with Snail1. Protein expression was determined in total cell extracts by western blot**. B,** Bone-marrow-derived macrophages were incubated for 48 h with

Tomato-labelled MCF7 cells and conditioned-medium (CM) from the indicated cells and the cytotoxic activity versus MCF7 cells was assayed. **C**, RNA analysis of CAF-regulated genes in not-stimulated macrophages or treated with CM from the indicated cells. Regulation of these same genes by CAFs (data from Fig 5) is shown as reference. In B-C the average ± SD of three experiments is shown. Statistical significance was obtained using Student's t-test; ns, not significant; *, P <0.05; ***, P <0.001.

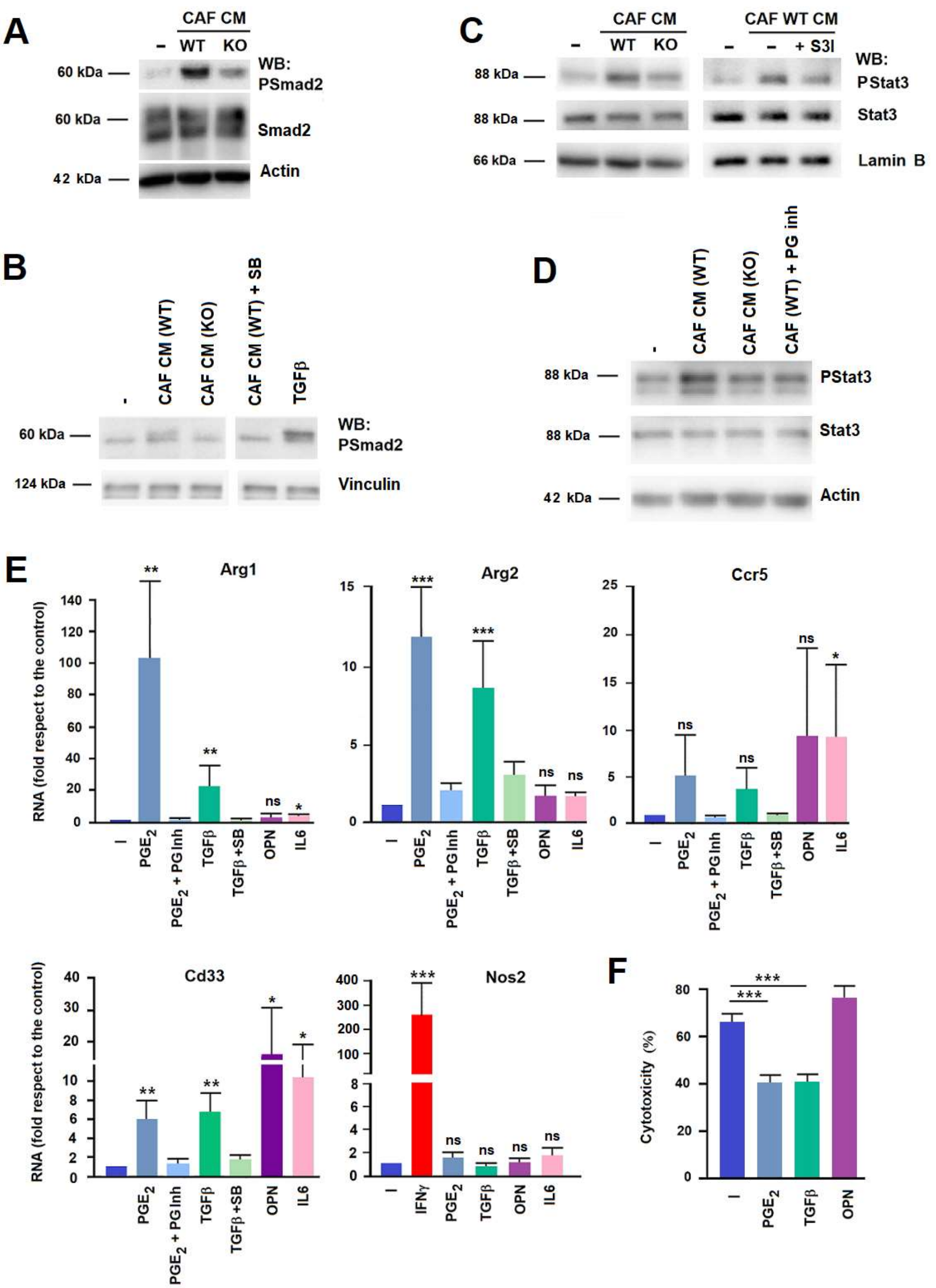

Figure S8. Prostaglandin E_2 (PGE_2) stimulates Arg1 and Arg2 gene expression and represses macrophage cytotoxicity. A-D, Smad2 or Stat3 phosphorylation was assessed by western blot in NMuMG (A) or bone-marrow-derived macrophages (BMDMΦ) (B-D) treated with conditioned medium from wild-type (WT) or Snail1-depleted (KO) CAFs. When indicated the Stat3 phosphorylation inhibitor S3I (25 μM),

the TGFβ-receptor inhibitor SB (5 μM) or the PGE_2 receptor inhibitors L161 (10 μM) and PF (1 μM) (PG Inh) were also added. **E**, BMDMΦ were treated with PGE_2 (100 nM), TGFβ (5 ng/ml), Osteopontin (OPN) (10 ng/ml), IL6 (50 ng/ml), PG Inh or SB. RNA was obtained after 24 h and the expression of the indicated genes was analyzed by real-time quantitative PCR coupled to retrotranscription. Average ± SD of three experiments is shown. **F**, BMDMΦ were incubated with the indicated factors and Tomato-labelled human MCF7 for 48 h. Macrophage-induced cytotoxicity was assessed as above. The figure shows the average ± SD of three experiments. In E and F statistical significance was obtained using Student's t-test; *, P <0.05; **, P <0.01; ***, P <0.001.

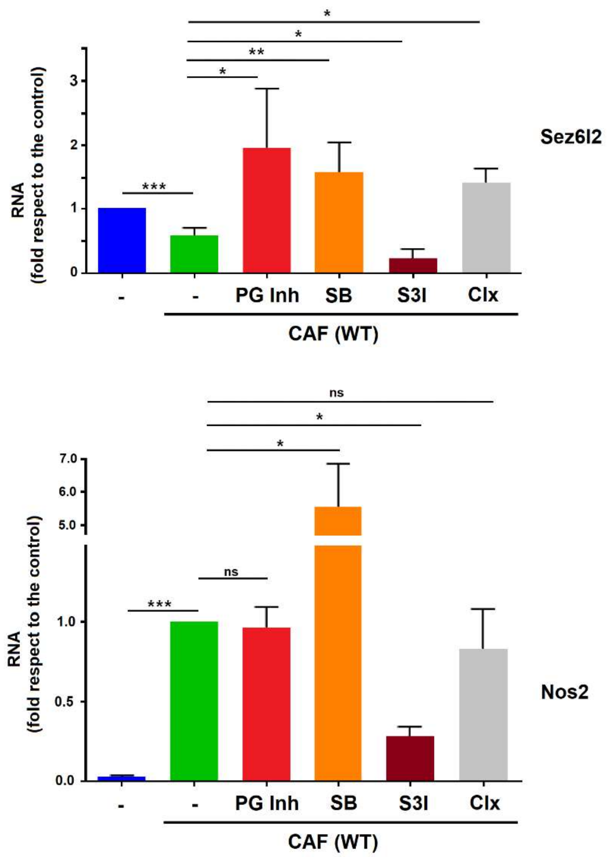

Figure S9. Prostaglandin E_2 (PGE_2) and TGFβ receptor inhibitors reverse the repression of *Sez6l2* by cancer-associated fibroblasts (CAFs)-stimulated macrophages. RNA analysis of macrophages treated with conditioned medium (CM) from CAFs. The indicated inhibitors were added to the CM. When the effect of celecoxib (Clx) was assayed, this compound was previously added to the CAFs during 24 h. The average ± SD of three experiments is shown. Statistical significance was obtained using Student's t-test; ns, not significant; *, P <0.05; **, P <0.01; ***, P <0.001.

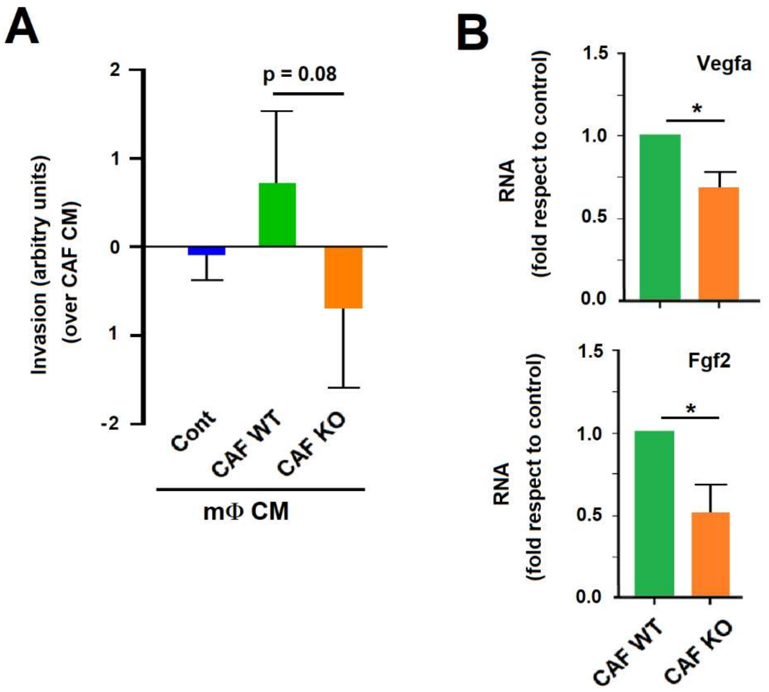

Figure S10. Functional effects of cancer-associated fibroblast (CAF)-induced macrophage polarization on endothelial cells. A, Conditioned medium (CM) from macrophages stimulated either by wild-type (WT) or Snail1-depleted (KO) CAFs CM was used to drive invasion of HMEC1 endothelial cells. CAFs CM was used as control. **B**, Expression of *Vegfa* or *Fgf2* genes was assessed in macrophages by real-time quantitative PCR coupled to retrotranscription. The figures show the average ± SD of three experiments. Statistical significance was obtained using Student's t-test; *, P <0.05.

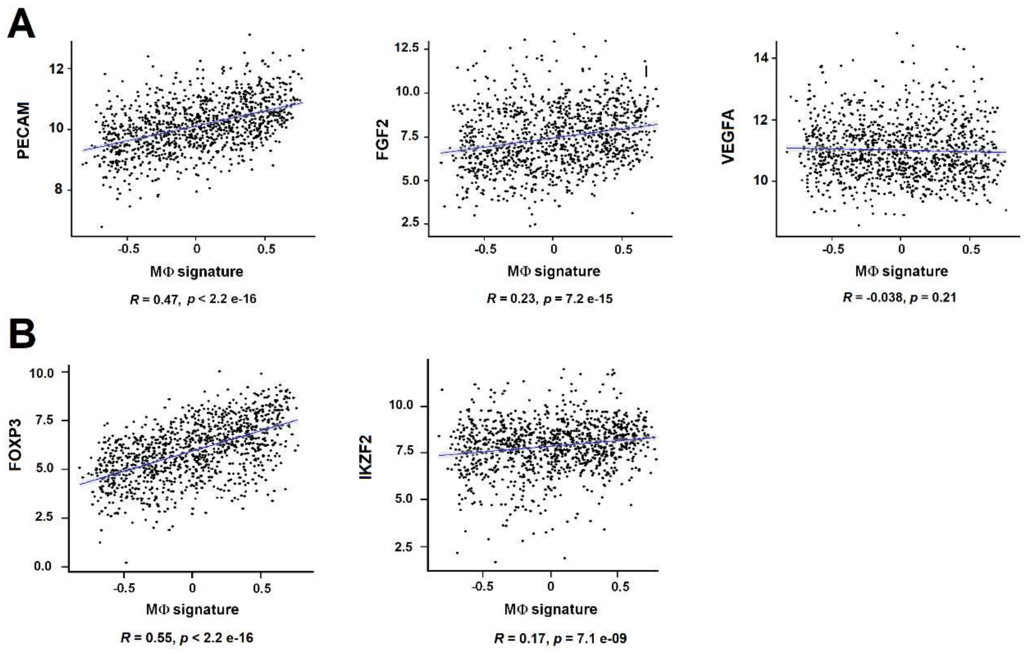

Figure S11. The macrophage gene signature specific for cancer-associated fibroblast (CAF)- activation correlates with endothelial cell markers and genes characteristic of activated regulatory T cells (T-regs). The signature characteristic of CAF-activated macrophages was analyzed using the GSVA method in TCGA Breast Invasive Carcinoma PanCancer Atlas dataset; Spearman's correlation analysis showed positive associations among this macrophage's signature (*PLXDC2/ TIMP1/ CCR5/ ARG2/ CD33/ CXCR4/ MRC1/ CCR1/ ARG1*) and endothelial cells-related genes (**A**) or with genes characteristic of activated T-regs (**B**). The blue line represents regression.

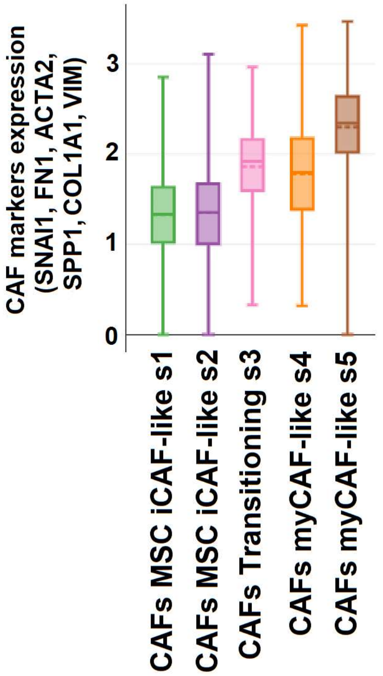

Figure S12. **Cancer- associated fibroblast (CAF)** ***myCAF-like s5*** **signature presents the highest association with CAF activation markers.** Breast cancer patients single cell RNA seq data analyzed online at https://singlecell.broadinstitute.org/single_cell/ portal/ as indicated in Methods. CAF populations were scored by their expression of the specific CAF markers analyzed in Fig. 8A (*SNAI1, FN1, ACTA2*, *SPP1*, *COL1A1* and *VIM*). Each box is defined between Q1 and Q3 values and includes a dashed line (median) and a solid line (average). The bars represent the maximum and the minimum value of the series.

Table S1. Antibodies used in this article

Antibody	Company	Method
α- Smooth Muscle Actin (α-SMA)	Sigma (A2547)	WB (1/1000)
β-Actin	Abcam (ab8227)	WB (1/2000)
Arginase 1	Cell Signaling (93668)	WB (1/1000)
Arginase 2	Cell Signaling (55003)	WB (1/1000)
CD3 (clone 17A2) (APC-Cy7)	BD Biosciences (560590)	FC (1/200)
CD4 (clone GK1.5) (PerCP)	Biolegend (100431)	FC (1/100)
CD8a (Clone 53-6.7) (PE-Cy7)	EBioscience (25-0081-82)	FC (1/100)
CD11b (clone M1/70) (PE-Cy7)	BD Biosciences (561098)	FC (1/100)
CD25 (clone 3C7) (APC)	Biolegend (101909)	FC (1/100)
CD45 (clone 30-F11) (PE)	Biolegend (103105)	FC (1/100)
CD69 (Clone H1-2F3) (PE)	Biolegend (104507)	FC (1/100)
CD206 (Mrc1)	Abcam (ab64693).	IHC (1/100)
E-Cadherin	Transduction labs (610182)	WB (1/2000)
F4/80 (clone BM8) (APC)	Biolegend (123115)	FC (1/100)
Fibronectin1	Abcam (ab2413)	WB (1/1000)
FoxP3 (Clone FJK-16s) (PE)	EBioscience (12-5773-82)	FC (1/200)
FoxP3	Cell signaling (12653)	IHC (1/200)
Lamin B	Abacam (ab16048)	WB (1/2000)
Ly6G (clone RB6-8C5) (FITC)	EBioscience (11-5931-81)	FC (1/100)
MHCII (clone M5/114.15.2) (APC-Cy7)	Biolegend (107627)	FC (1/100)
pSmad2	Cell Signaling (3108)	WB (1/1000)
pStat3	Cell Signaling (9145S)	WB (1/1000)
Pyruvate kinase	Chemicon (1235)	WB (1/1000)
Smad2/3	Cell Signaling (8685)	WB (1/1000)
Snail1	Cell Signaling (3879)	WB (1/2000)<br>IHC (1/100)
Stat3	Santa Cruz (Sc-8019)	WB (1/1000)
Tubulin	Merck/Sigma (T9026)	WB (1/10000)
Vimentin	BD Biosciences (550513)	IHC (1/50)
Vinculin	Sigma (V9131)	WB (1/1000)

WB: western blot; IHC: immunohistochemistry; FC: flow cytometry

Table S2. Primers used in the real-time quantitative PCR coupled to retrotranscription.

Gene	Forward	Reverse
Arg1	5'-CACACTGACATCAACACTCC-3'	5'-TCTCGCAAGCCAATGTACAC-3'
Arg2	5'-TCTCCTCCACGGGCAAATTC-3'	5'- GCAAGCCAGCTTCTCGAATG-3'
Ccl2	5'-GCATCTGCCCTAAGGTCTTCA-3'	5'GTGGAAAAGGTAGTGGATGCATT-3'
Ccr5	5'-GCTCAGTCTTTCCTGCATGGT-3'	5'-ACTTCCTAAGCTCCCACTGC-3'
Cd33	5'-TCCATTCATGCTTTTCTTAGCTGT-3'	5'-GAGTTAGGACGCATTTTAGCTGT-3'
Cd86	5'- CAGCACGGACTTGAACAACC-3'	5'- CTCCACGGAAACAGCATCTGA -3'
Ciita	5'-AGGCCTATGCCAACATTGCG-3'	5'-CCATAGCATGCTCTTCCGGG-3'
Fgf2	5'-GACCCACACGTCAAACTACAA-3'	5'-GTAACACACTTAGAAGCCAGCAG-3'
H2-Aa	5'-AGGTGAAGACGACATTGAGG-3'	5'-AACTCAGGAAGCATCCAGAC-3'
H2-Ab	5'-CCATTACCTGTGCCTTAGAG-3'	5'-GAACTGGTACACGAAATGCC-3'
H2-Dmb1	5'-AGCCTTCTCCAGCGTTTGC-3'	5'-TTTGGGCTACTCGGACAGATG-3'
Hprt	5'-GGCCAGACTTTGTTGGATTTG-3'	5'-TGCGCTCATCTTAGGCTTTGT-3'
Ido1	5'-AACCCTATAAGAGATGCTGGGACAT-3'	5'-ATGTGGTAGAGCAAAGCCCA-3'
Itgax	5'-GAGGCTGCAAGCATCATTCG-3'	5'-GCATCAAAGTTCTCCACGCTG-3'
Mrc1	5'-CTGCAGATGGGTGGGTTATT-3'	5'-GGCATTGATGCTGCTGTTATG-3'
Msr1	5'- ATCTTCCACCAAGGCCAGTG-3'	5'- CCTAGACTCCGGCAGACAAC -3'
Myc	5'ATGAGGAGACACCGCCCACC-3'	5'-GCCTCTTCTCCACAGACACCACAT-3'
Nos2	5'-AGCTGGGCTGTACAAACCTT-3'	5'-CTCCCATGTTGCATTGGAAG-3'
Opn	5'-CTGGCAGCTCAGAGGAGAAG-3'	5'-CTGTGGCGCAAGGAGATTCTG-3'
Sez6l2	5'-GACGCAGAGGGAAAGTGACA-3'	5'-TTCCAGGCTCTGGCATCATC-3'
Snai1	5'-GCGCCCGTCGTCCTTCTCGTC-3'	5'-CTTCCGCGACTGGGGGTCCT-3'
Snai2	5'-CCATGCCATCGAAGCTGAGAA-3'	5'-AGGCTTTTCCCCAGTGTGAGTTC-3'
Tgfb1	5'-AAGTGGAGCAGCACGTGGAG- 3'	5'-CAGCCGGTTGCTCAGGTATC-3'
Thbs1	5'-AAGCAACCGCATTCCAGAG-3'	5'-TGGCCCTTCACCAGTCG-3'
Twist1	5'-CTGCAGGCCGGAGACCTAGA-3'	5'-TTAAAAGTGTGCCCCACGCC-3'
Vegfa	5'-CCAGCGAAGCTACTGCCGTC-3'	5'-GATCCGCATGATCTGCATGG-3'
Zeb1	5'-TCAGCTGCTCCCTGTGCAGT-3'	5'-AAGGCCTTCCCGCATTCAGT-3'

All sequences correspond to the murine genes.

Table S3. Secreted factors differently expressed in wild-type (Snail WT) versus Snail-depleted (Snail1 KO) cancer-associated fibroblasts

PROTEIN	FOLD CHANGE (Snail1 WT vs Snail1 KO)
IGFBP-7	653.41
CCL27	422.62
Osteopontin	202.02
Osteoprotegerin	22.74
TIMP-1	21.90
CCL17	11.92
CXCL1	10.77
G-CSF	10.10
Spinesin Ectodomain	6.91
CCN3	6.60
MCP-1	5.17
CXCL16	5.01
MFG-E8	4.94
IL-22	4.69
IL-15	4.54
VEGF	4.44
CCL25	4.40
IL-24	3.95
Fractalkine	3.92
Progranulin	3.89
Granzyme G	3.89
Urokinase	3.84
TGFβ	3.61
Granzyme D	3.55
MCP-5	3.51
MIG	3.37
IL-6	3.33
RAGE	3.31
TFPI	3.27
Fas Ligand	3.21
Activin A	3.20
PDGF C	3.15
CCL7 / MCP-3 / MARC	3.00
Lymphotactin	2.98
MMP-3	2.90
IL-13	2,78
IGF-I	2.72
TCA-3	2.69
IL-7	2.67
IL-21	2.65
Galectin-3	2.58
GM-CSF	2.54

Dkk-3	2.51
Gremlin	2.46
RANTES	2.43
VCAM-1	2.43
CCL8 / MCP-2	2.42
IL-3	2.39
TRAIL / TNFSF10	2.35
CCL22	2.34
TGF-beta 3	232
S100A10	2.29
CXCL5	2.15
SLPI	2.15
HGF	2.13
CD14	2.12
DPPIV / CD26	2.11
FGF-21	2.07
Thymus Chemokine-1	2.07
RANKL	2.05
MIP-1 gamma	2.03
IL-4	2.01
IL-22BP	1.99
FADD	1.97
Cerberus 1	1.95
GDF-3	1.90
FLRG (Follistatin)	1.90
Ubiquitin	1.87
PlGF-2	1.87
TSLP	1.84
CD30 L	1.83
Artemin	1.80
MIP-3 alpha	1.80
Activin C	1.79
Thrombospondin	1.78
IL-23	1.78
MIP-3 beta	1.76
TGF-beta 2	1.73
IL-20	1.72
IL-1 beta	1.71
Decorin	1.71
TL1A / TNFSF15	1.70
CCL4 / MIP-1 beta	1.69
MIP-1 alpha	1.69
Fit-3 Ligand	1.69
CCR7	1.69
IL-18 R alpha/IL-1 R5	1.69
DAN	1.68

Resistin	1.67
IL-10	1.67
CXCR4	1.66
CCR3	1.66
IFN-alpha / beta R2	1.65
IL-31 RA	1.65
BCMA / TNFRSF17	1.65
TIMP-4	1.64
WIF-1	1.64
ALCAM	1.63
Insulin	1.61
CCL1 / I-309 / TCA-3	1.60
TROY	1.60
TACI / TNFRSF13B	1.58
Axl	1.57
CD27 / TNFRSF7	1.56
IFN-beta	1.55
Neg	1.55
HVEM / TNFRSF14	1.53
IL-2 R beta	1.51
GDF-5	1.51
IL-17RD	1.51
Crossveinless-2	1.50
Chordin-Like 2	1.50
IL-17BR	1.50
IL-12 p40/p70	1.49
Eotaxin	1.48
IL-2	1.47
Frizzled-1	1.46
IL-9 R	1.45
LEPTIN(OB)	1.44
Shh-N	1.42
GDF-1	1.40
I-TAC	1.40
BTC (Betacellulin)	1.40
Angiopoietin-like 3	1.40
EDAR	1.39
VEGF-D	1.37
IL-15 R alpha	1.37
Angiopoietin-like 2	1.37
CCR4	1.36
Activin RIB / ALK-4	1.36
beta-Catenin	1.35
CXCR3	1.33
Cripto	1.33
DKK-1	1.32

SCF	1.31
IL-17D	1.31
Eotaxin-2	1.31
Granzyme B	1.30
MMP-14 / LEM-2	1.30
GDF-9	1.30
Endoglin / CD105	1.29
ICK	1.28
MMP-24 / MT5-MMP	1.27
TIMP-2	1.26
RELM beta	1.24
Cryptic	1.23
b FGF	1.21
MMP-9	1.21
TLR1	1.21
IFN-alpha / beta R1	1.20
BAFF R / TNFRSF13C	1.20
Coagulation Factor III / Tissue Factor	1.18
EG-VEGF / PK1	1.17
IFN-gamma	1.16
6Ckine	1.16
VE-Cadherin	1.16
uPAR	1.16
CD40 Ligand / TNFSF5	1.15
IGFBP-6	1.15
MMP-12	1.15
IL-23 R	1.14
FAM3B	1.14
CD27 Ligand / TNFSF7	1.14
TPO	1.13
VEGFC	1.12
OX40 Ligand / TNFSF4	1.12
MMP-2	1.12
M-CSF	1.11
IL-17C	1.11
Pentraxin3 / TSG-14	1.06
Lefty-1	1.02
Serum Amyloid A1	1.00
BLC	1.00
CRP	1.00
IGFBP-5	0.98
SDF-1	0.98
IGF-II	0.96
TWEAK / TNFSF12	0.96
Epigen	0.96
PF-4	0.96

CRG-2	0.93
Adiponectin / Acrp30	0.92
CD11b	0.90
Neuregulin-3 / NRG3	0.89
IL-9	0.88
IL-27	0.87
Dkk-4	0.86
Prolactin	0.85
IL-11	0.85
IL-17E	0.84
IGFBP-1	0.83
IL-17	0.82
LIF	0.81
Kremen-1	0.81
Cardiotrophin-1	0.79
Epiregulin	0.78
IL-28 / IFN-lambda	0.78
IGFBP-3	0.76
MIP-2	0.75
TNF-alpha	0.74
Follistatin-like 1	0.72
GITR Ligand / TNFSF18	0.71
CCL28	0.69
AgRP	0.68
Lungkine	0.68
IL-31	0.67
Erythropoietin (EPO)	0.66
GDF-8	0.66
IL-5	0.65
LIGHT / TNFSF14	0.65
Endocan	0.65
CXCL14 / BRAK	0.64
TNF-beta / TNFSF1B	0.63
VEGF-B	0.61
Endostatin	0.61
IGFBP-2	0.60
Neurturin	0.58
IL-1 alpha	0.56
IL-16	0.52
SPARC	0.46
IL-12 p70	0.39
IL-17F	0.35
WISP-1 / CCN4	0.34
Soggy-1	0.31
